# Disentangling thermal stress responses in a reef-calcifier and its photosymbionts by shotgun proteomics

**DOI:** 10.1038/s41598-018-21875-z

**Published:** 2018-02-23

**Authors:** Marleen Stuhr, Bernhard Blank-Landeshammer, Claire E. Reymond, Laxmikanth Kollipara, Albert Sickmann, Michal Kucera, Hildegard Westphal

**Affiliations:** 1 Biogeochemistry and Geology, Leibniz Centre for Tropical Marine Research (ZMT), 28359 Bremen, Germany; 2 Leibniz-Institut für Analytische Wissenschaften – ISAS – e.V., 44139 Dortmund, Germany; 3 Medizinische Fakultät, Medizinische Proteom-Center (MPC), Ruhr-Universität Bochum, 44801 Bochum, Germany; 4 Department of Chemistry, College of Physical Sciences, University of Aberdeen, Aberdeen, AB24 3FX Scotland United Kingdom; 5 MARUM, Center for Marine Environmental Sciences, University of Bremen, 28359 Bremen, Germany; 6 Department of Geosciences, University of Bremen, Bremen, Germany

## Abstract

The proliferation of key marine ecological engineers and carbonate producers often relies on their association with photosymbiotic algae. Evaluating stress responses of these organisms is important to predict their fate under future climate projections. Physiological approaches are limited in their ability to resolve the involved molecular mechanisms and attribute stress effects to the host or symbiont, while probing and partitioning of proteins cannot be applied in organisms where the host and symbiont are small and cannot be physically separated. Here we apply a label-free quantitative proteomics approach to detect changes of proteome composition in the diatom-bearing benthic foraminifera *Amphistegina gibbosa* experimentally exposed to three thermal-stress scenarios. We developed a workflow for protein extraction from less than ten specimens and simultaneously analysed host and symbiont proteomes. Despite little genomic data for the host, 1,618 proteins could be partially assembled and assigned. The proteomes revealed identical pattern of stress response among stress scenarios as that indicated by physiological measurements, but allowed identification of compartment-specific stress reactions. In the symbiont, stress-response and proteolysis-related proteins were up regulated while photosynthesis-related proteins declined. In contrast, host homeostasis was maintained through chaperone up-regulation associated with elevated proteosynthesis and proteolysis, and the host metabolism shifted to heterotrophy.

## Introduction

Most marine reef-building organisms such as corals rely on symbiosis with photosynthesizing microalgae. Tropical coral reef ecosystems are one of the structurally most complex and richest hotspots of biodiversity on Earth^1^. The symbiotic relationship provides the holobiont with a clear advantage in nutrient–limited settings, but it comes at a cost of lower resilience to perturbations^2,3^. Indeed, marine photosymbiotic ecosystems are threatened and decline rapidly due to human activity. Global warming is currently considered as the most damaging factor^4^, inducing bleaching responses in reef organisms, i.e. the loss of photosynthetic microalgae and/or photo-pigments^5,6^. Massive bleaching events are reported more frequently, already becoming a regular occurrence^4,7^. Ocean warming rarely affects marine ecosystems directly by elevated mean seawater temperature. Instead, heat stress is often induced during episodic heating events. Subsequently, research on thermal tolerance of marine photosymbiotic organisms has shifted from determining thermal limits under constant exposure to the consideration of different thermal stress frequency scenarios. Studies on photosymbiotic corals and large benthic foraminifera (LBF), i.e. photosymbiont-bearing calcifying eukaryotes, have demonstrated that, compared to chronic stress, transient heat-stress events have little immediate impact on these organisms^8–10^. On the contrary, variable temperature regimes might even increase their resilience towards warming, because fluctuations may facilitate acclimatization and promote recovery from heat stress events^8,11^. The possible energetic costs and underlying molecular mechanisms of this acclimatization^11^ are important aspects that need to be understood in order to make projections on adaptive capacity and resilience potential of coral reef organisms.

The most direct approach to reveal the molecular mechanisms of resistance and adaptation to thermal stress involves the analysis of proteome composition. In symbiont-bearing organisms the stress response involves two compartments (host and symbiont) and appropriate methods are needed to disentangle the specific proteome responses^12^. Due to high sensitivity and high throughput at relatively low costs, “omics” approaches have advanced quickly, providing promising new insights in the molecular mechanisms of stress responses in symbiont-bearing marine organisms^13–15^. Whilst proteomics provides powerful tools to understand how stress affects the biology of organisms, there are limitations to its application. Mass spectrometry based proteomics is strongly dependent on the coverage of sequence databases, which can be especially challenging in environmental research and limits its applications to well-studied model organisms. This bottleneck can be circumvented by performing cross-species homology searching by hybrid *de novo* peptide sequencing and database search approaches^16,17^. Furthermore, previous studies have focused on non-symbiotic diatoms^18–20^ or only the host proteomes^15,21^, while invertebrate and algae proteomes are rarely analysed simultaneously. This is possible by performing proteomics analysis of holobionts and annotating the peptides/proteins *in silico* to either host or symbionts^14,22^. By targeting photosymbiotic organisms as a functional unit, including the host and the endosymbiotic algae, key interactions between both compartments can be detected.

A suitable organism to test this approach and reveal compartment specific molecular response to thermal stress are symbiont-bearing benthic foraminifera, because the collection and cultivation of statistically significant sample sizes is easy and efficient, and with minimal impact on local reef resources^23^, but most molecular techniques are challenging to apply such that novel approaches are of high interest^24^. The circum-global genus *Amphistegina*, which inhabits oligotrophic coral reef environments, hosts diatom photosymbionts^25^ and is a vital constituent of coral reef ecosystems^26^. Due to their physiological sensitivity^27^ to environmental changes they are commonly used as bioindicators for past and present coral reef health^23,28,29^ and provide a useful model to study the effects of environmental change on photosymbiotic calcifiers. Their algal symbionts enable these calcium carbonate producers to generate approximately 3.9–5.4% of reef carbonate sediments^30^. Both chronic thermal stress and high light intensities can induce bleaching in LBF, which is usually accompanied by a multitude of other afflictions and can ultimately diminish populations and reduce carbonate accumulation^9,31–36^.

Long-term heat stress appears to affect LBF primarily by disturbing the photosynthetic performance of the symbionts^32,33,36,37^, causing reduced holobiont calcification and growth^9,35–37^ and reducing host activity^9,32^. In contrast, they display a marked capacity for acclimatization to short-term thermal stress events that do not induce bleaching^9^ but the exact mechanisms of acclimatization and thermal stress response remain unresolved. Existing protein expression studies revealed decreases in the rate-limiting carbon fixation enzyme ribulose 1-5-biphosphate carboxylase/-oxygenase (RuBisCO)^38^ and high ratios of the 70 kDa stress protein^39^ in response to heat shocks. These gel-based approaches can only target specific proteins, are challenging to apply to small protein volumes and do not allow partitioning between host and symbionts^24^.

In order to reveal insights into their mechanisms of response to ocean warming, we carried out an experiment comparing three thermal stress scenarios^9^. Here, *A. gibbosa* populations were exposed to (a) no thermal stress i.e. control conditions at constant 25.5 °C, (b) a single temporary thermal stress event of three days up to 32 °C followed by control conditions, (c) episodic temporary thermal stress events alternating with periods of six days at control conditions and (d) chronic thermal stress at 32 °C over one month (Supplementary Fig. S1). Alongside quantification of classical physiological response parameters that were previously discussed in an accompanying publication by the authors^9^, a subset of specimens from the same experiment has been used for a label-free proteome analysis by liquid chromatography-tandem mass spectrometry (LC-MS/MS), allowing simultaneous evaluation of the host and symbiont compartments. The experimental setup combining proteomics with physiological measurements allows us to (i) authenticate the dual-compartment approach on non-model organisms, (ii) elucidate response mechanisms induced by single and episodic thermal stress, and (iii) determine the underlying molecular response to chronic thermal stress, including LBF bleaching.

## Results and Interpretation

### Dual-compartment Protein Identification

In this study, a total of 1,618 proteins belonging to the concatenated host-symbiont database were identified by homology-driven search approaches, all of which were present in samples of all treatments and at the beginning of the experiment. In order to condensate these protein sequences – stemming from closely related organisms – a similarity-based clustering step was performed, which condensed 1,618 protein sequences to 1,136 protein clusters (Supplementary Table S3). The largest cluster comprised 10 protein sequences (all related to various actin isoforms), while 926 proteins remained as single-protein clusters. Out of all protein clusters, approximately 31% were assigned to the host foraminifera and 68% to the symbiont compartment. The proportion of the proteins assigned to each compartment should reflect the ratio of symbiont to host biomass. This has never been determined for foraminifera, but considering the observed symbiont density, the ratio is likely to be about balanced. A confounding factor, however, is the coverage of the protein database. This is much poorer in foraminifera, so the expected sign of effect would be to underestimate the amount of foraminifera proteins. Since the ratio we observe is 1:2 in favour of the symbionts, the database coverage effect is likely occurring, but it does not seem to be large (order of magnitude). Nine protein clusters contained sequences stemming from both host and symbionts and could not be clearly associated to a compartment, likely because the respective protein sequences are highly conserved. These ambiguous clusters have been removed from further analyses. Protein clusters will be simply referred to as proteins in the following sections.

### Multivariate Analysis of Relative Protein Abundance Changes

**Figure 1:**
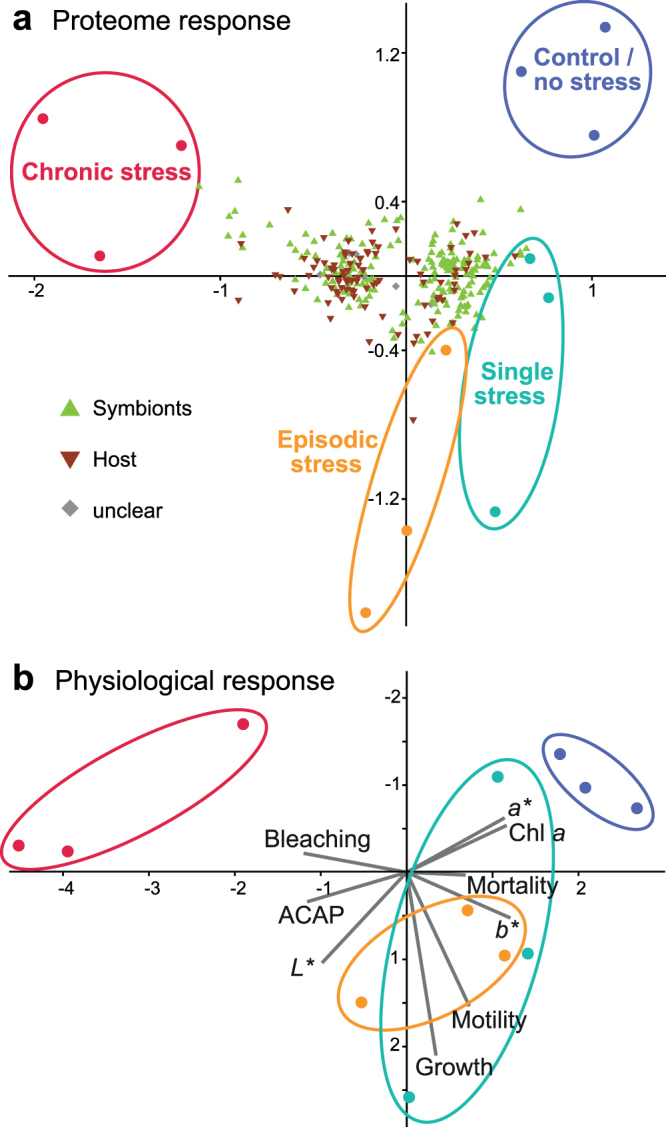
Comparison of proteomics results with the previously published physiological response of *Amphistegina gibbosa* in response to a single short-term temporary stress event (turquoise), episodic temporary stress events (orange) or chronic thermal stress (red) compared to the control treatment (blue). (**a**) Correspondence analysis of relative protein abundances of all 294 regulated proteins, showing the distribution of proteins (host = brown inverse triangles, symbiont = green triangles, unclear/both = grey diamonds) that drive the directional changes between treatments. 65.8% are explained by variation along axis 1 and 11.2% by axis 2. (**b**) Principal component analysis of physiological metrics of LBF from the same experiment^9^, including chlorophyll *a* concentration (Chl *a*), total capacity against peroxyl radicals (ACAP), motility, growth rate, CIE L*a*b* colour space values (L* = whiteness, a* = green to magenta, b* = blue to yellow), mortality and bleaching frequency. 60.1% are explained by variation along principal component 1 and 18.6% by principal component 2.

The level of change in protein abundances infers different effects of elevated water temperatures on *Amphistegina gibbosa*. Significant variations between treatments were observed, particularly under chronic thermal stress (Fig. 1a). These directional proteome developments in different treatments were characterized by the trend and amount of change in abundance of regulated proteins (*p*-ANOVA ≤ 0.05). The proteome responses are largely in agreement with the physiological variables investigated within the same experiment^9^ and therefore demonstrate high congruency of the outcomes of both approaches (Fig. 1b). This is understood as verification of the credibility of our dual-compartment proteomics approach. Additionally, future availability of transcriptome or whole genome sequencing data could further boost the sensitivity of our proteomic analysis and be used to re-analyse the obtained data to gain further insights not attainable by homology-based search methods. Furthermore, continually increasing sensitivity enables the application of protein and peptide fractionation methods even for minute sample amounts^40^, which can increase the depth of further proteomics analyses of LBF.

**Figure 2:**
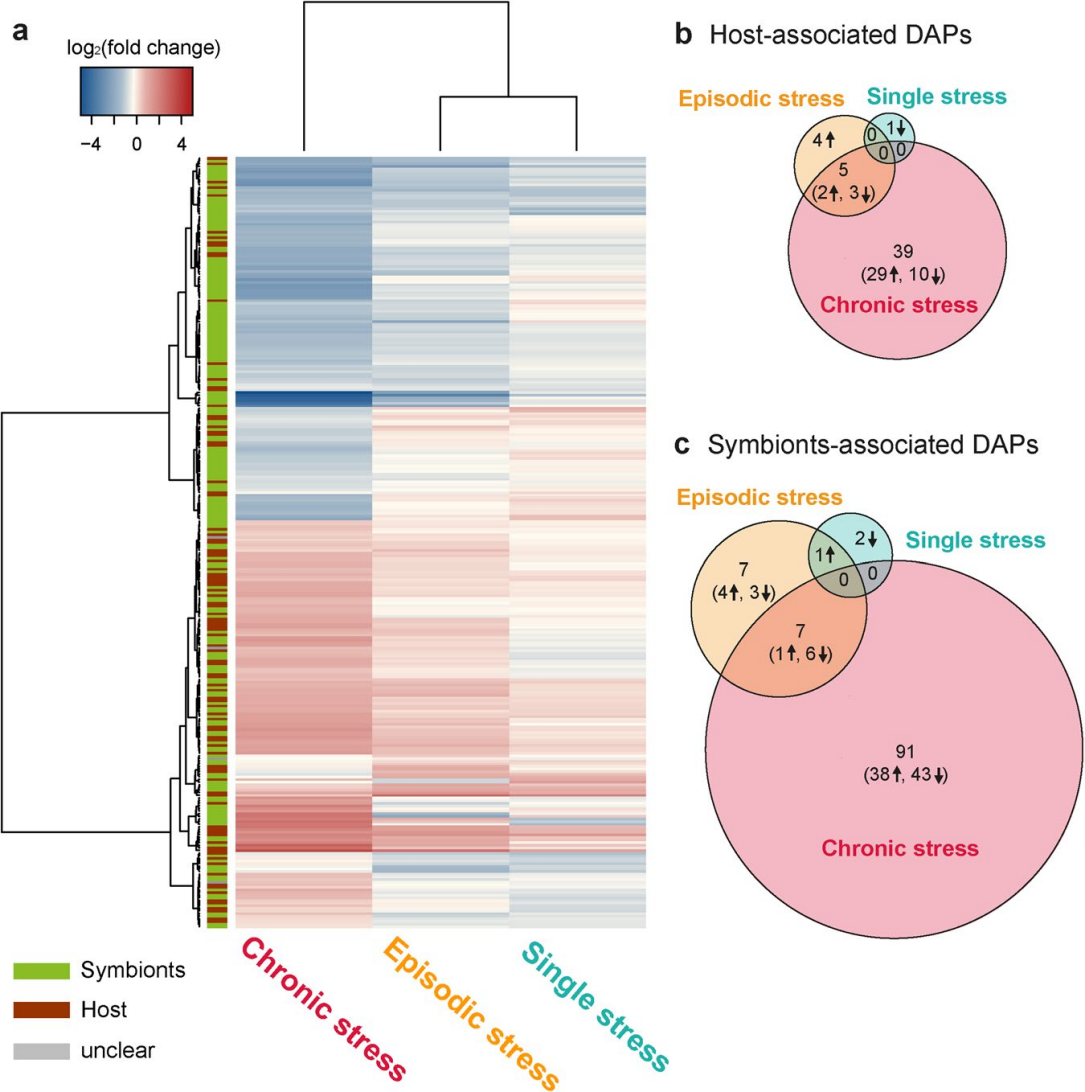
The heatmap with hierarchical cluster analysis (Euclidean distance) of all regulated proteins compared to the control (**a**) illustrates the direction of log_2_ fold changes (FC) in protein abundances and their distribution among host foraminifera (brown) and symbionts (green) in *Amphistegina gibbosa*. The normalised abundance values, significant *p-*values, and accessions of all regulated protein clusters are reported in Supplementary Table S3. Venn diagrams show the amount of proteins in *A. gibbosa* that significantly changed in abundance (DAPs) in response to a single stress event (turquoise), episodic stress events (orange) and chronic stress (red) compared to the control in (**b**) the host (n = 49) and (**c**) the symbiont compartment (n = 108). Overlapping areas show protein groups that were equally regulated in more than one treatment. Arrows indicate how many proteins were up (↑) or down (↓) regulated.

More than 25% of all identified proteins were regulated with respect to the control (Fig. 2a, Supplementary Figs S2, S3). The small extent of regulation between the single-stress treatment and only slightly altered development in response to episodic stress, in contrast to the discrete impact of chronic-stress treatment, reflects the LBF holobionts’ capability to maintain or quickly restore biological functions/homeostasis during or after temporary thermal stress events. In contrast to the temporary-stress treatments, LBF exposed to chronic stress underwent distinct changes, indicating that the nature of the proteome response to thermal stress is depending on the persistence of stress exposure. The analysis illustrates that those proteins assigned to the host compartment are mostly distributed in direction of the chronic-stress treatment (Supplementary Fig. S3). The foraminifers’ reaction hence plays a more prominent role in continuous stress exposure, while proteomic changes in response to temporary-stress treatments took place dominantly in the symbiont compartment.

With respect to the start condition (Supplementary Fig. S7), the LBF proteomes in all treatments evidently underwent changes. The specimens at the start of the experiment were in the adult stage, close to maximum size, and we have observed reproduction in some of the specimens^9^. It is possible that physiological changes in the foraminifera occur prior to reproduction. This could account for the observed drift in the proteome composition of the control. However, we are reluctant to attribute the observed drift in the control population solely to this effect. This is because we do not know how long the foraminifera require for acclimation to culture conditions and that the observed drift could reflect on-going acclimation. Notwithstanding the exact mechanism, the observed drift does not introduce a random effect into our test design, because it shifted the composition of the proteomes between the start and end in all replicates equally (Table S3). We consider it nevertheless prudent to document the effect and the pertinent data and figures with respect to start conditions are available in the supplementary material (Table S3 and Figs S8–S10).

### Proteomic Responses to Thermal Stress

To gain more precise insights into the most important cellular mechanisms occurring in response to the different thermal-stress scenarios, we here now focus on the differently abundant proteins (DAPs), i.e. those regulated proteins that could be clearly assigned to one of both compartments, showed significant variations between treatments in Tukeys’ HSD post hoc test (*p*-value ≤ 0.05) and a distinct positive or negative change in abundance (log_2_ FC > 1 or < −1). With these stringent criteria, 49 proteins in the host (Fig. 2b) and 108 proteins of the symbiont compartment (Fig. 2c) were differentially abundant in comparison to the control. They are summarized in the Supplementary Tables S1 and S2, where they are organized by log_2_ fold changes and colour-coded by general biological functions and processes. These are not exclusive but rather describe their most prominent general cellular roles. The complete list of gene ontology (GO) term annotations, including assignment of molecular functions, biological processes and cellular components, is given in the Supplementary Table S4.

The single temporary-stress treatment only induced four DAPs in total. The three-day stress peak at the beginning of the experiment thus had very little influence on the holobiont proteome in the long-term. Both compartments showed more pronounced responses in reaction to episodic stress events. In the symbiont compartment, the increased DAPs belonged to protein folding and degrading categories that are typically increased in response to stress in order to maintain homeostasis. Depleted DAPs incorporated some proteins participating in photosynthesis and other carbohydrate metabolizing processes, indicating a slight reduction of carbon concentrating mechanisms. Overall, a minor stress response was detected, but no lethal impacts on the diatoms derived from the episodic stress events. Interestingly, some processes in the host seemed to be slightly stimulated by the thermal fluctuations. Although actin, serine peptidase, and serine palmitoyltransferase were depleted, suggesting that the cytoplasm might have been damaged, the actin-related protein 2 was increased along with few metabolic and biosynthesis-related proteins. Since this protein mediates actin nucleation, new cytoskeletal filaments were likely created and/or actin-cytoskeleton based processes like cell locomotion, phagocytosis or intracellular motility of vesicles were enhanced^41^. These results are at odds with the proteomic responses of corals that show down-regulation of Hsps as well as proteins involved in translation and metabolic processes when exposed to quarter-daily temperature fluctuations^14^. Yet our previous observations reflect the proteomic outcome as holobiont motility and growth rates were also highest in this treatment^9^. Whether the foraminifers activity was enhanced as a general stress reaction, for repair or acclimatization, or if thermal variations act positively on the holobionts performance could not be determined here. However, a fast reaction to the recently experienced thermal fluctuations could be crucial for their resilience to environmental variations.

### Compartment-specific Impacts of Chronic Thermal Stress

**Figure 3:**
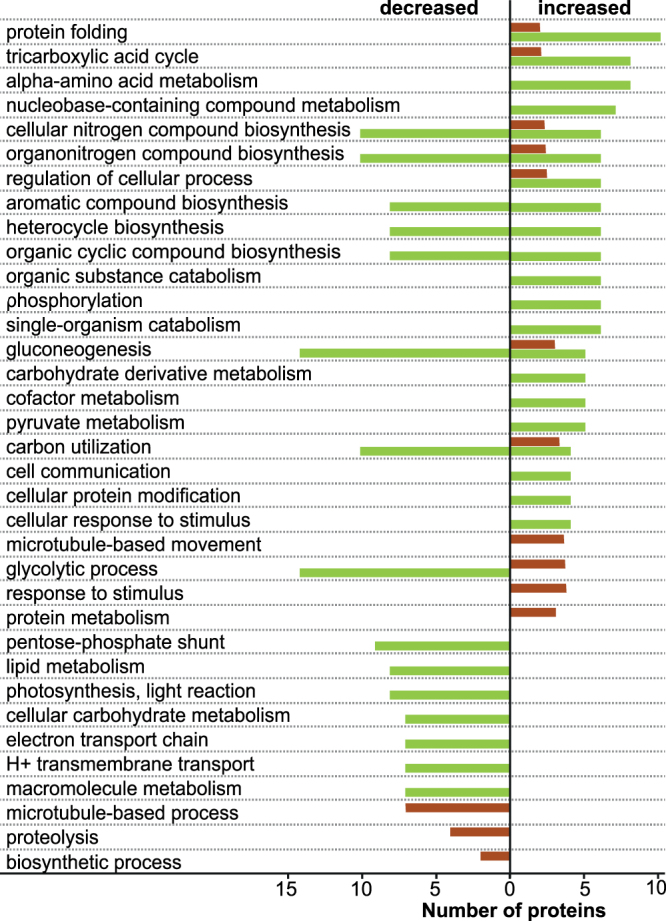
Counts of differently abundant proteins in *Amphistegina gibbosa* in response to chronic thermal stress in the host (brown) and the symbiont (green) compartment grouped by biological process gene ontology terms. Position of bar indicates if proteins were more (right side) or less (left side) abundant than in the control.

The most and strongest changes in protein abundances of both compartments were induced by chronic thermal stress, impacting a multitude of functions and processes (Fig. 3, Supplementary Figs S4–S6). Together, these suggest that the following cellular responses and changes in pathways occurred in the chronic-stress treatment:

#### Symbionts – Oxidative Stress, Protein Folding and Degradation

Heat stress in algae usually leads to the production of reactive oxygen species (ROS) by a malfunctioning of the photosystems, generating oxidative stress that may severely damage the cells^6^. Our detection of significantly elevated DAPs that are usually responsible for protein quality control, folding and degradation (e.g., Hsps, calreticulin, prohibitin) affirm that protein and cell damage occurred. Predominantly, members of the Hsp70 family were elevated, corresponding to the temperature-induced increase in Hsp gene expression in diatoms described previously^42^. The production of these chaperones, which directly mediate correct folding and bind to partially denatured proteins to prevent their aggregation, is usually stimulated by proteotoxic stress, but also the synthesis of new proteins requires chaperones for assistance in folding^43^. Here, we also detected a remarkable up-regulation of proteins responsible for cell death and degradation such as the hypersensitive-induced response^44^ and an autophagy-related protein (Atg8)^45^, indicating programmed cell death. Hypersensitive response is a mechanism that in plants is usually observed as a final stage in reaction to pathogens that leads to the rapid death of infected cells, possibly triggered by the presence of ROS^44^. Atg8 is an ubiquitin-like protein that is essential for the formation of autophagosomes, which sequester bulk cell materials to be degraded and deliver them to the vacuoles, but it is also thought to play a key role in selective autophagy^45^. Moreover, calreticulin/calnexin, which binds to defective or incorrectly folded proteins and thereby targets them for degradation, and at least one member of the Clp proteases family^46^ that often act in selective proteolysis^47^ were elevated. Similarly, the impairment of the photosynthetic electron transport and respiratory chains in iron deprived diatoms results in excess ROS accumulation, likely inducing redox-sensitive mechanisms that regulate metabolic rates in order to ensure cellular homeostasis^18^ or triggering programmed cell death^20^.

**Figure 4:**
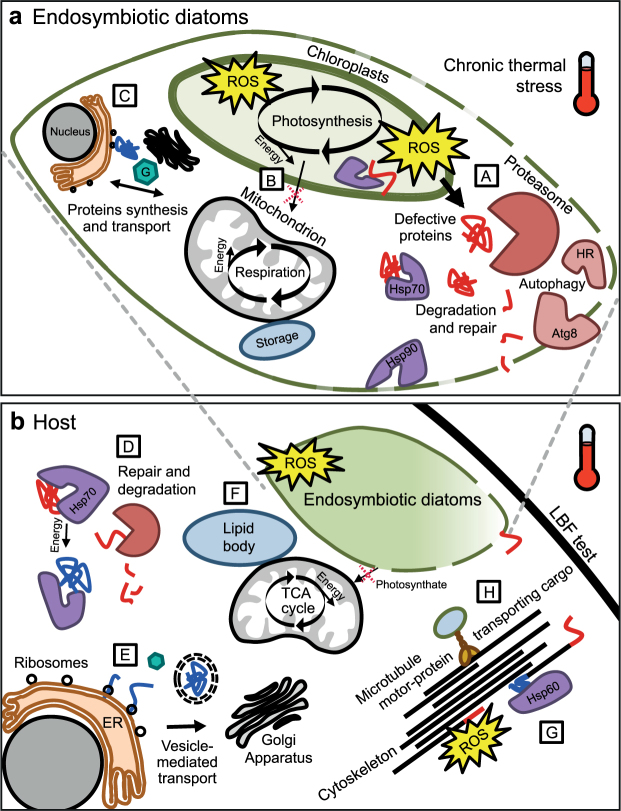
Chronic thermal stress induced the proposed cellular processes in the (**a**) photosymbionts and (**b**) the host cell of the large benthic foraminifera *Amphistegina gibbosa*. (A) Malfunctioning photosystems in the diatom chloroplasts produce reactive oxygen species (ROS), which damage cellular compounds. Molecular chaperones (purple) such as Hsp70 and Hsp90 stabilize and repair defective proteins (red) or target them for degradation by the proteasome. Cell death-related proteins (rose) such as hypersensitive response (HR) and autophagy-related protein 8 (Atg8) disassemble cellular components. (B) The damaged photosystems produce less cellular energy and photosynthate. To compensate for this and meet metabolic requirements, alternative resources e.g. stored in form of lipids are catabolized by the mitochondria. (C) Cellular constituents are partly reorganized through enhanced ion transport and cellular signalling ‘G’ proteins in combination with augmented biosynthesis of proteins (blue), possibly recycling the degraded compounds. In the host (**b**), the ROS lead to damaged or incorrectly folded proteins (D), but cellular homeostasis is maintained by chaperone-modulated repair mechanisms and targeted protein degradation. (E) Cell cycle-related functions of the endoplasmic reticulum (ER), ribosomes and the Golgi such as protein synthesis and modification combined with increase in proteins responsible for transport and signal conduction facilitates enhanced protein turnover. (F) Energy resources stored in lipid bodies are metabolized, e.g. through the tricarboxylic acid (TCA) cycle, to meet the augmented metabolic needs necessary for these stress response mechanisms, despite diminished supply of photosynthate by the impaired symbionts. (G) The cytoskeleton is damaged by the ROS-induced stress (red ends), but increased amounts of chaperonins such as Hsp60 assist in folding and stabilization of new cytoskeletal proteins. (H) Microtubule motor-proteins such as dynein increase to enhance intracellular transport, which could enable the host foraminifera to enhance heterotrophic feeding, transport of (damaged) proteins or symbionts to their required location, and the reallocation of energy storages.

In contrast to an expected up-regulation of antioxidants, the only recognized peroxidase (phospholipid methyltransferase) detected in the symbiont compartment was strongly depleted. This suggests that the amount of oxygen radicals might have surpassed the available ROS scavengers and could thus have damaged lipids, proteins and DNA^6^. Interestingly, we found a severe reduction of HopJ type III effector proteins (hypersensitive response and pathogenicity-dependent outer protein), which usually play key roles in the host-pathogen interactions in the type III secretion system of pathogenic bacteria^48^. These pathogens cause diverse diseases in hosts, based on their ability to colonize the intercellular spaces of plant tissues and cause death. As effector proteins usually help pathogen to invade host tissue and suppress its immune system, analogous functioning proteins might be crucial to keep the symbiosis in LBF intact, i.e. to protect the diatoms inside the host cell from being destroyed. Considering possible interactions that might cause the observed degradation of endosymbionts during bleaching in LBF, such mediators should be studied in more depth. Overall, the strong expression of chaperones and degradation-related proteins shows that exposure to heat did not only induce repair mechanisms, but also cell death and protein degradation in the photosymbionts. As we observed severe bleaching of our heat stress specimens^9^, this is in line with prior studies^9,18,32^ and shows that prolonged thermal stress considerably harms the performance and induces deterioration of diatom symbiont cells (Fig. 4a).

#### Symbionts – Reduction of Carbon Fixation and Core Metabolism

Because of their evolutionary history, diatom metabolism pertains some exceptional features in comparison to other photosynthetic eukaryotes (e.g. green algae and land plants), such as the presence of enzymes more commonly found in prokaryotes and the possession of the enzymatic machinery required for C4 carbon fixation^49^. Additionally, the mode of CO_2_ concentration and the posttranslational regulation of photosynthetic products are highly complex and remain largely unknown among diatoms^50^. In our study, the symbiont-associated DAPs that decreased strongest comprised a variety of chloroplastic proteins and enzymes involved in photosynthesis and the coupled carbon-fixating metabolism (e.g. RuBisCO, fucoxanthin chlorophyll *a/c*). Carbohydrate metabolic processes, specifically the production of polysaccharides via photosynthesis and the Calvin cycle, seemed to be severely impaired by the damaging effects of chronic heat exposure. Consequently, also DAPs participating in conversion of the resulting carbohydrates into storage compounds^49^ or their utilization via glycolysis or parallel metabolic pathways^50^ appeared to be diminished. These pathways represent major sources of cellular energy (mostly stored and transported in the form of ATP) and provide the precursors that are necessary for the synthesis of many essential biomolecules such as lipids. The deficiency of CO_2_ concentrating mechanisms was hence similarly detectable by the severe reduction of chloroplastic ATPases and other proton transporters.

Although proteomic responses of diatoms to several environmental stressors have been studied^18,20^ and temperature was identified as the major driver influencing diatom growth^19^, no comparable studies have focused on endosymbiotic living diatoms. In the planktonic diatom *Pseudonitzschia multiseries*, elevated temperature leads to enhanced intracellular protein transport and turnover^19^. Other studies determined optimal temperature for the model diatom *Thalassiosira pseudonana* to be around 25 °C, with growth and photosynthetic performance clearly dropping at higher temperatures^42^. Likewise, elevated temperatures have been shown to lead to decreased photosynthetic efficiency in other LBF^32,37^ and might even induce photoinhibition^34^. Heat shock experiments on the diatom-bearing LBF *Baculogypsina sphaerulata* demonstrated significant decreases in the expression of RuBisCO at 34 °C, but not at temperatures up to 32 °C^38^. As days to weeks of exposure to sub-lethal heat can severely decrease photosynthetic efficiency^32,37^, our results support the hypothesis that the heat-induced reduction in RuBisCO may diminish carbon fixation^38^ seen through reduced calcification and holobiont growth^9,32,35,36^. The observed depletion of photopigment concentrations and clearly decreasing but still on-going holobiont oxygen production measured within the same experiment^9^ further support these findings and highlight the severe consequences of thermal stress on proteins responsible for the generation of carbohydrates and cellular energy.

#### Symbionts – Adjustment of Resource Management and Cell Cycle

In order to maintain the cellular energy metabolism and provide carbon to downstream metabolic pathways during reduced photosynthesis, glucose produced by degradation of storages such as polysaccharides and lipids needs to be increasingly catabolized via glycolic processes and the tricarboxylic acid (TCA) cycle. Correspondingly, some enzymes that are typically taking part in carbohydrate, lipid and amino acid metabolism were elevated under chronic thermal stress. The DAPs included enzymes of different respiratory pathways and even enzymes involved in photorespiration such as the glyoxylate cycle (aconitase hydratase 2)^50^, which bypasses the CO_2_-generating steps of the TCA cycle. The increase of acyl-CoA dehydrogenase, which initiates and catalyses the rate-limiting step in the conversion of lipids to acetyl-CoA, showed an enhanced lipid catabolism. Besides, we found small GTPases, GTP- and ATP-binding proteins to have highly increased in abundance. These are involved in signal transduction, ion transport (e.g. Ca^2+^-ATPase), but also play roles as sources of energy or activators of substrates in metabolic reactions and are used for protein synthesis and gluconeogenesis. Augmented degradation of lipids and carbohydrates through glycolysis and the TCA cycle leads to elevated production of amino acids and other important biomolecules. As also cell cycle-related proteins increased, biosynthetic processes and cellular reorganization might have been supported, which likely required enhanced signal transmission, compound and energy supply. In diatoms grown under suboptimal condition of nutrient or iron limitation, alike shifts from proteins related to photosynthetic carbon fixation to proteins involved in cellular respiration^20^ and ribosomal translation^19^ were observed. Jointly, the observed symbiont-associated DAPs in our study suggest that the stress response and degradation processes of the symbionts came along with the reduction of their CO_2_-concentrating mechanisms, leading to a reduction of energy storage and reorganization of other cellular constituents, possibly by recycling the remaining compounds through alternative metabolic pathways^50^.

#### Host – Protein Production, Folding and Degradation

Opposite to the symbionts, more than twice as many DAPs were enriched than reduced in the host compartment. The majority of the elevated proteins likewise included heat shock proteins and other chaperones or constituents involved in protein folding and quality control as well as proteolysis (Fig. 4b). These outcomes confirm the results of an Hsp70 immunoblotting study on the non-symbiotic benthic foraminifera *Ammonia tepida*^39^, which showed that elevated temperatures induce a significant increase in heat shock proteins in foraminifera, as it does under stressful conditions^43^ in most organisms. At lethal temperatures, no foraminiferal Hsp70 is detectable anymore^39^. Therefore, although foraminifera ceased to move or grow in our chronic-stress treatment^9^, the abundance of host Hsps show that the treatment was not lethal yet. Besides a variety of stress proteins (i.e. Hsp70/90 family, chaperonin, calreticulin, prohibitin and ubiquitin), a 26S protease regulatory subunit and caseinolytic peptidase (Clp), were elevated. These peptidases regulate intracellular protein levels as well as the turnover of defective proteins^46^. They thereby contribute to maintaining the cellular proteostasis in selectively removing damaged or incorrectly folded proteins^47^. Specifically the 26S proteasome is responsive to oxidative stress and was equally enhanced in anemones under heat stress^21^. This seems to be at odds with the observation that another serine peptidase (S10) was depleted, which specifically cleaves small peptides instead of full-length proteins^46^. Its deficiency might indicate a shift of proteolysis from decomposing smaller biomolecule remains to concentrating on whole protein degradation in order to maintain homeostasis.

The stress induced boost of protein turnover was further indicated by enhanced cell cycle related functions. Extreme increases in abundance were found in a RNA helicase (DEAD box polypeptide 46) and a translation initiation factor that are implicated in the alteration of RNA cellular processes necessary for protein synthesis, cellular growth and division. Furthermore, two signalling small GTPases (cell division control 42 and Ypt1) were increased. The former can interact with multiple regulators and effectors to activate a variety of cellular processes, mostly pathways leading to actin rearrangements and transcriptional inductions^51^. Ypt1 regulates the trafficking of secretory vesicles from the endoplasmic reticulum (ER) to the Golgi, conducted by the trafficking particle complex (TRAPP)^52^, of which one subunit was also elevated. Jointly, the augmentation of proteins involved in the translation, transport and modification of proteins, together with simultaneous elevated abundance of molecular chaperones and regulatory proteins, indicate an intensification of cell cycle-related processes in the foraminifera that kept the cellular homeostasis of the host intact.

#### Host – Metabolic Adjustments

Maintaining homeostasis in response to thermal change also requires shifts in metabolism to accommodate changes in the organism’s energy requirements or of the flux of different metabolite classes. The strongest DAP decrease in the host was found for a phosphoethanolamine *N*-methyltransferase-like enzyme participating in the metabolism of glycerophospholipids, the main components of biological membranes. This is in contrast to heat shocked anemones that showed a strong increase in this enzyme^21^. A reason might be an inhibition of *S*-adenosyl-L-methionine (SAMe) synthesis through the methionine cycle, because enzymes depending on this primary donor of methyl in eukaryotic cells were depleted in both compartments. Another reduced enzyme (serine palmitoyltransferase) is essential for the biosynthesis of sphingolipids, which may serve vital functions in cell biology. The activity of this housekeeping enzyme is regulated in diverse ways and was suggested to be increased during apoptosis in response to certain types of stress^53^, while the lowered abundance observed in our study might as well be due to deficiencies of essential constituents.

The up-regulated metabolic changes were more pronounced, including a wide range of proteins involved in core carbon metabolism. Assuming that significantly less photosynthate was released from the impacted photosymbionts to the host, metabolic needs have had to be met by adjusting pathways from mainly relying on carbohydrates supplied by the diatoms to digesting energy stored e.g. in lipid droplets. Similar to the symbiont compartment, an increase of cellular respiration was observed, specifically among enzymes catabolizing glycolysis (and gluconeogenesis), which represents a highly conserved response to cellular stress. Previous studies^33^ on the ultrastructure of *A. gibbosa* describe significant declines of lipid bodies along with reduced numbers of viable symbionts during LBF bleaching. We therefore hypothesize, specifically with regard to the high energetic requirements for stress response and repair mechanisms, that elevated TCA cycle activity and digestion of lipids and other storage molecules compensate for the photosynthate deprivation in order to meet the host’s metabolic demands. Since the foraminifera were fed in the cultures and thus had an external carbon source, it is difficult to discern whether the metabolism of the foraminifera switched to more heterotrophy from the external source or also by utilizing the biomass of the symbionts.

#### Host – Contradicting Trends of Cytoskeletal Proteins

Actin and tubulin constitute the cytoskeleton and represent the majority of proteins found in foraminifera^41^. Under chronic thermal stress, both were strongly depleted. These biomolecules fulfil diverse partly overlapping cellular functions (e.g. cell migration, adhesion and division). While actin builds filaments and plays a role in membrane trafficking, tubulin builds the microtubules that participate in the control of protrusive and contractile forces. Both compounds interact in multiple ways, such as in cell motility^41^ i.e. movement of the holobiont within the vial, which accordingly is severely impaired in LBF by chronic thermal stress^9,32^. Likewise, former cytological studies revealed deterioration of the host cytoplasm under combined stress of high temperature and light^33^. Similar processes have likely occurred to a minor extent in our experiment. Actin filaments are highly sensitive to oxidative stress and were equally impacted in thermally shocked *Aiptasia*^21^. As in anemones, the combination of reduction in cytoskeletal proteins with increases in molecular chaperones and proteins responsible for translation and transport of new biomolecules indicate a replacement of the lost proteins.

Counter to the reduction of cytoskeleton-building proteins, proteins involved in microtubule-based movement (e.g. dynein) increased in response to chronic heat exposure. This suggests that the host raised its microtubule-motor activity in order to enhance cytoplasmic transportation of particles as would be necessary for more heterotrophic feeding or greater motility^41^. Phototaxic studies have shown that LBF seek shade through reticulopodial locomotion when exposed to high light intensities^27^, which might also be caused by high temperatures. Furthermore, it was recently shown that actin-mediated relocation of symbionts in LBF plays a key role in photoprotective mechanisms^54^, moving symbionts away from the high light causing photic stress. Another reason might be that, as suggested for corals^55^, the importance of heterotrophic feeding in order to meet nutritional demands was increased during bleaching, requiring high cytoskeletal activity to collect and transport food particles into the endoplasm. Since specimens exposed to chronic stress showed severe bleaching, lowered chlorophyll *a* concentrations, and cessation of movement at the end of the experiment^9^, we conclude that microtubule-activity was enhanced for intensification of intracellular transport, potentially enabling enhanced suspension feeding, transport of (damaged) proteins or symbionts targeted for degradation, or the reallocation of stored energy.

## Discussion

Our dual-compartment quantitative proteomics approach sheds light into the complex mechanisms responsible for the repair, translation and degradation of cellular functions during different heat stress exposure scenarios. The general proteome response pattern is similar to the observed physiological parameters (Fig. 1), confirming that the presented novel approach worked for the analysis of small-sample amounts where both symbiotic compartments cannot be physically separated and the host has only low sequence coverage. Additionally, our proteomics approach revealed the cellular mechanisms underlying these distinct reactions. The data denotes that the endosymbiotic diatoms were impacted more severely by elevated temperature than the host foraminifera. The symbiont proteome indicates reduced ability to photosynthesize, whereas the foraminifera show a signature of metabolic adjustment in favour of heterotrophy, compensating the less of symbiont-derived metabolites. This is in line with former studies suggesting that the host foraminifera is more resistant towards heat than their photosymbionts^9,34^.

We confirm the physiological experiment in that the strongest response is seen in chronic stress, whereas fluctuating water temperatures slightly impacted the photosymbionts, resulting in elevated activity in the host. The molecular mechanisms underlying the strong chronic-stress response involve degradation of defective compounds and repair of cellular damage, which is likely caused by ROS (Fig. 4). The thermal stress severely obstructed the functioning of the symbiotic diatoms, as observed in the detected photopigment loss and extensive bleaching. Increases in cell death and repair-related proteins indicate the disruption of proteostasis, and at the same time carbon concentrating mechanisms and transport of their products diminished. Foraminifera-associated proteins responsible for microtubule-based movement were strongly increased, along with molecular chaperones and holobiont total antioxidant capacities^9^. Thus, remaining resources were likely distributed from cell migration and growth towards stress response and the cellular reorganizations of the host. During the constant heat exposure of 30 days, these protective mechanisms, e.g. unfolded protein response and rapid protein turnover, apparently facilitated the LBFs survival. Key to this relatively high stress resistance could be the high nutritional flexibility of foraminifera, feeding on the photosynthate of their endosymbionts, storing energy in lipid droplets and the possibility to additionally feed heterotrophically, and should therefore be studied in more detail. Furthermore, bleaching in LBF has often been reported in combination with other impacts or diseases (such as reproductive dysfunction, infestations and malformations)^28,31^. These likely represent secondary effects or long-term consequences of resource redistribution. Such potential trade-offs deserve further investigation by targeted proteomic studies.

This study demonstrates the applicability of label-free proteomics on a non-model symbiotic organism and illustrates that the presented method offers novel opportunities to simultaneously study both compartments of photosymbiotic organisms, providing detailed insights into proteome responses and their effects on molecular functions. The successful application of the approach on a group with unusually poor molecular database coverage indicates that proteome analysis as implemented in this study may help to reveal the mechanisms of ecological response, biotic interactions and ecosystem-relevant functions in a range of similar organisms.

## Methods

### Thermal Stress Experiment

The experimental thermal-stress treatments implemented in this study were discussed in detail in previous work^9^. *Amphistegina gibbosa* were collected in 18 m depth at Tennessee Reef in the Florida Keys (24°45′8.33″N, 80°45′26.33″W). The LBF were brought to the laboratory in Bremen, Germany, and maintained at established culture conditions (25.5 °C, 5–10 µmole photons m^−2^s^−1^ on a 12-h light/dark cycle) for three weeks prior to the experiment (Supplementary Fig. S1a). During the entire time they were kept in 18-l aquaria filled with synthetic seawater (salinity 35.5, Tropic Marin Sea Salt, Germany) and equipped with a temperature sensor and a titanium heating rod to automatically control the thermal conditions, as well as an aquarium pump to circulate the water to simulate natural flow conditions (all from Aqua Medic, Germany). Temperature was logged constantly (HOBO Pendant, Germany), while salinity, pH and temperature were additionally measured manually every other day.

Within each of the 12 independent, randomly allocated aquaria, ten *A. gibbosa* specimens were kept in a glass vial, covered with a 400 µm-nylon mesh, which allowed the water to circulate into the vial, while keeping the LBF inside. The initial population included specimens > 0.6 mm, which is consistent with adult stage, not far from maximum size. Whilst the foraminifera were in an ontogenetically comparable stage throughout the experiment, the population of the symbionts likely underwent several generations of cellular division. Intracellular lifecycles of diatom symbionts in their foraminiferal host have been observed^56^, but never quantitatively characterised. In their free-living stage, most diatoms have fast cell cycles of hours to several days^57^, and if they do so also in the endosymbiotic state, the different temporary stress events in our experiment may have hit (partly) different generations.

Since the studied foraminifera are mixotrophic and require external carbon source, they were fed 15 µl of autoclaved microalgae^32^ every nine days. After three weeks of acclimation, the experiment ran for 30 days with three replicates of each of the four different thermal treatments simulating: (i) no thermal stress at culture conditions to serve as control; (ii) a single 3-day temporary thermal stress event (up to 32 °C) followed by stable control condition; (iii) four episodic 3-day temporary thermal stress events (up to 32 °C) intermitted by six days at control conditions; and (iv) chronic thermal stress at 32 °C (Fig. S1b). The temperature in all stress treatments was raised by ~0.25 °C per hour for the first 24 hours of the experiment to reach 32 ± 0.5 °C. While this temperature was kept constant in the chronic-stress treatment, it was only kept this high for one day in the temporary-stress treatments and then let drop back to control temperature. The response variables of the *A. gibbosa* holobiont (growth, motility, respiration) and their photosymbionts (photosynthesis, coloration, chlorophyll *a* content) were documented on days 0, 3, 12, 21, and 30 in order to monitor the temporal variations in their physiological performance^9^.

At the start of the experiment, i.e. after acclimation, five subsamples of ten specimens each were taken as initial controls. By aid of a fine paintbrush, the specimens were very shortly placed on a filter paper to remove the circumjacent water, directly transferred into cold 1.5 ml Protein LoBind tubes (Eppendorf, Germany), and immediately frozen at –80 °C until further processing. The same procedure was performed with all samples at the end of the experiment.

### Proteome Analysis

Eight specimens were pooled per experimental condition and biological replicate in 1.5 ml Eppendorf tubes, resulting in 15 sample pools. As 24 specimens per treatment and the start condition were analysed, 120 specimens were used in total. After addition of 100 µl lysis buffer (LB), comprised of 50 mM Tris-HCl (pH 7.8), 150 mM NaCl, 1% SDS and Complete Mini, lysis was carried out by mechanical grinding (Fig. S1c). After storage on ice for 30 minutes, samples were clarified by centrifugation at 4 °C and 10,000 rcf for 10 min.

Protein concentration was estimated based on reference samples of which the exact protein concentration was determined by amino acid analysis as previously described^58,59^. Cysteines were reduced by the addition of DTT to a final concentration of 10 mM and incubation at 56 °C for 30 min. Subsequently free thiol groups were alkylated with 30 mM IAA at room temperature (RT) for 30 min in the dark.

Buffer exchange and proteolysis were carried out by an adapted filter-aided sample preparation^60,61^ workflow. Lysates corresponding to an approximated protein concentration of 9 µg were diluted 5-fold by addition of freshly prepared^62^ 8.0 M Urea/100 mM Tris-HCl (pH 8.5) and transferred onto the centrifugal device (PALL Nanosep, 30 kDa cutoff). Centrifugation was carried out at RT for 30 min at 13,500 rcf and all following centrifugation steps were performed under the same conditions for 15 min. Three wash steps were carried out with 100 µl of 8.0 M Urea/100 mM Tris-HCl (pH 8.5). To exchange the buffer, the centrifugal devices were washed three times with 100 µl of 50 mM NH_4_HCO_3_ (pH 7.8). To the concentrated proteins, 100 µL of proteolysis buffer comprising trypsin (1:20 w/w ratio of protease to substrate), 0.2 M GuHCl and 2 mM CaCl_2_ in 50 mM NH_4_HCO_3_ (pH 7.8) was added and samples were incubated at 37 °C for 14 h. Digested peptides were recovered by centrifugation followed by consecutive washing steps with 50 µL of 50 mM NH_4_HCO_3_ and 50 µL of ultra-pure water. The digestion was stopped by addition of 20 µl of 10% TFA. Digests were desalted using SPEC C18, 4 mg sorbent (Agilent) as per manufacturers’ instructions, and quality-controlled as described previously^63^.

First, aliquots of each sample corresponding to ~1 µg of peptides were analysed on a nano-LC-MS system in order to compensate for systematic errors derived e.g. from the protein concentration estimation. Thus, the sample amounts were corrected based on the alignment of total ion chromatograms to warrant identical starting material prior to actual LC-MS analysis. After normalization of amounts, all twelve samples (each 1 µg) were analysed using an Ultimate 3000 nano RSLC system coupled to a Q Exactive HF mass spectrometer (both Thermo Scientific, Fig. S1d). Peptides were preconcentrated on a 100 µm × 2 cm C18 trapping column for 10 min using 0.1% TFA with a flow rate of 20 µL/min followed by separation on a 75 µm x 50 cm C18 main column (both PepMap RSLC, Thermo Scientific) with a 120 min LC gradient ranging from 3–35% of buffer B: 84% ACN, 0.1% FA at a flow rate of 250 nL/min. The Q Exactive HF was operated in data-dependent acquisition mode and MS survey scans were acquired from m/z 300 to 1,500 at a resolution of 60,000 using the polysiloxane ion at m/z 371.101236 as lock mass^64^. Isolation of precursors was performed by the quadrupole with a window of 0.4 m/z. The fifteen most intense signals (Top15) were subjected to higher energy collisional dissociation with a normalised collision energy of 27% at a resolution of 15,000, taking into account a dynamic exclusion of 12 s. Automated gain control target values were set to 3 × 10^6^ for MS and 5 × 10^4^ MS/MS. Maximum injection times were 120 ms and 250 ms, respectively. Precursor ions with charge states of +1, > +5 or unassigned were excluded from MS/MS analysis. The “underfill” ratio, which specifies the minimum percentage of the target value likely to be reached at maximum fill time, was defined as 5%, which corresponds to a minimum precursor intensity of 2.5 × 10^3^ to trigger a MS/MS scan. The mass spectrometry proteomics data have been deposited to the ProteomeXchange Consortium via the PRIDE^65^ partner repository with the dataset identifier PXD008015.

The following chemicals were obtained from Sigma Aldrich, Steinheim, Germany: anhydrous magnesium chloride (MgCl_2_), guanidine hydrochloride (GuHCl), iodoacetamide (IAA), ammonium bicarbonate (NH_4_HCO_3_) and urea. Sodium chloride (NaCl) and calcium chloride (CaCl_2_) were from Merck, Darmstadt. Sodium dodecyl sulfate (SDS) was bought from Carl Roth, Karlsruhe, Germany. Tris base was purchased from Applichem Biochemica, Darmstadt, Germany. Dithiothreitol (DTT), EDTA-free protease inhibitor (Complete Mini) tablets were obtained from Roche Diagnostics, Mannheim, Germany. Sequencing grade-modified trypsin was bought from Promega, Madison, WI USA. All chemicals for ultra-pure HPLC solvents such as formic acid (FA), trifluoroacetic acid (TFA) and acetonitrile (ACN) were obtained from Biosolve, Valkenswaard, the Netherlands.

### Data Analysis

Data analysis for label free quantification was performed using the Progenesis LC-MS software (version 3.0.6039.34628) from Nonlinear Dynamics (Newcastle upon Tyne, U.K.) and the statistical software R, version 3.3.1^66^. Biological triplicates from all treatments at the end of the experiment were compared to the start condition, and for further insights, biological triplicates of single-, episodic- and chronic-stress treatments were compared to the control (Fig. S1e).

Raw MS data was imported into Progenesis and aligned to one automatically selected LC-MS reference file. After peak picking, MS/MS spectra were exported as peak list and identification of proteins and peptides was performed by the PEAKS Studio software suite, version 7.5, *de novo* to SPIDER^67^. Searches were performed in a decoy-fusion manner against a concatenated database comprising publicly available protein and nucleotide sequences of foraminifera as well as diatoms (Bacillariophyta) from the NCBI database on 2016/03/15. Protein sequence databases were used as such, while nucleotide sequences were translated in all six reading frames prior to concatenation. Precursor mass tolerance was set to 10 ppm and fragment ion tolerance to 0.02 Da. Enzyme specificity was set as fully tryptic, with a maximum of two missed cleavages. For *de novo* and DB search, carbamidomethylation of cysteines was defined as fixed modification and oxidation of methionine as variable modification. A maximum number of two variable modifications per peptide was allowed for *de novo* and DB search, while for the PTM search all common modifications were allowed (485 in total) and the maximum number was set to three per peptide. Minimum *de novo* ALC for both PTM and SPIDER homology search was set to 15%. Peptide-level FDR was limited to 1% and proteins had to be identified with at least one unique peptide in order to be reported. Identifications meeting these criteria were re-imported into Progenesis to calculate the normalised abundances on the peptide-level.

Amino acid sequences of all proteins identified in the database search were uploaded in.fasta file format to the CD-HIT suite web server of the Weizhong Li Lab^68^ in order to generate cluster of homologous protein sequences. Sequence identity cut-off was set to 0.7 and minimum alignment coverage for the longer sequence was set to 0.0. All other parameters were left as default. Normalised abundance-values of the peptides were used to calculate protein cluster abundance (Fig. S1f). Only peptides unique to a given protein cluster were used for quantification, hence, peptides that could not be clearly assigned to either of the compartments (host or symbionts) were removed from further analysis.

To identify statistically significant changes of protein abundance between the conditions, first the one-way between-subjects ANOVA was calculated. For regulated proteins, i.e. proteins with a *p*-ANOVA ≤ 0.05, Tukeys’ HSD post hoc test was performed to determine the statistical significance for every individual condition compared to the control. Regulated protein clusters with a Tukeys’ HSP post hoc test *p*-value ≤ 0.05 and a log_2_ fold change (FC) greater than 1 or below –1 were considered as significantly changed in abundance. Hierarchical cluster analysis, using Euclidean distance matrices, were computed and plotted as heatmaps for proteins and treatments by the functions dist and hclust in R^66^, and dendrograms were generated based on Ward’s minimum variance method.

For all sequences that significantly changed in abundance in any of the thermal-stress treatments, we performed an additional gene ontology annotation using Blast2GO, version 4.1.5^69^ to assess the protein identification coverage of our workflow and sequence databases (Fig. S1g). All sequences were concatenated and searched against all eukaryotes in the non-redundant (nr) public NCBI database using blastp and a maximum E-value of 1.0 × 10^–5^ reporting only the three top Blast hits. This was followed by the mapping function to assign gene ontology (GO) terms to each sequence with Blast hits. The GO term annotations were evaluated by the Blast2GO annotation rule algorithm using the following criteria: E-value filter 1.0 × 10^–6^, annotation cutoff 55 and GO weight 5. To complement the functional information of the proteins, an InterPro scan of these sequences was performed and GO information was merged. The resulting best Blast hits were adopted as sequence descriptions. If no specific protein names could be identified by the Blast search, available InterPro protein names were included. Based on the major molecular functions, biological processes and cellular component annotations, the proteins were grouped into different functional categories. These are not exclusive, but rather generic as many proteins can fulfil a multitude of functions and therefore might also fit into different categories. The entire annotation results can be found in the electronically available Supplementary Table S4.

## Electronic supplementary material

Supplementary Information

Table S3

Table S4
