# Supplementary material for: Disentangling thermal stress responses in a reef-calcifier and its photosymbionts by shotgun proteomics
Source: Sci Rep. 2018 Feb 23;8:3524. doi: 10.1038/s41598-018-21875-z (PMC5824892; doi:10.1038/s41598-018-21875-z)
Supplement: Supplementary file 1 — Supplementary Information [file 41598_2018_21875_MOESM1_ESM.pdf]

Disentangling thermal stress responses in a reef-calcifier and its photosymbionts by shotgun proteomics

Marleen Stuhr[1*], Bernhard Blank-Landeshammer[2], Claire E. Reymond[1], Laxmikanth Kollipara[2], Albert Sickmann[2,3,4], Michal Kucera[5], Hildegard Westphal[1,6]

[1] Biogeochemistry and Geology, Leibniz Centre for Tropical Marine Research (ZMT), 28359 Bremen, Germany

[2] Leibniz-Institut für Analytische Wissenschaften – ISAS – e.V., 44139 Dortmund, Germany

[3] Medizinische Fakultät, Medizinische Proteom-Center (MPC), Ruhr-Universität Bochum, 44801 Bochum, Germany

[4] Department of Chemistry, College of Physical Sciences, University of Aberdeen, Aberdeen AB24 3FX, Scotland, United Kingdom

[5] MARUM, Center for Marine Environmental Sciences, University of Bremen, 28359 Bremen, Germany

[6] Department of Geosciences, University of Bremen, Bremen, Germany

* marleen.stuhr@leibniz-zmt.de

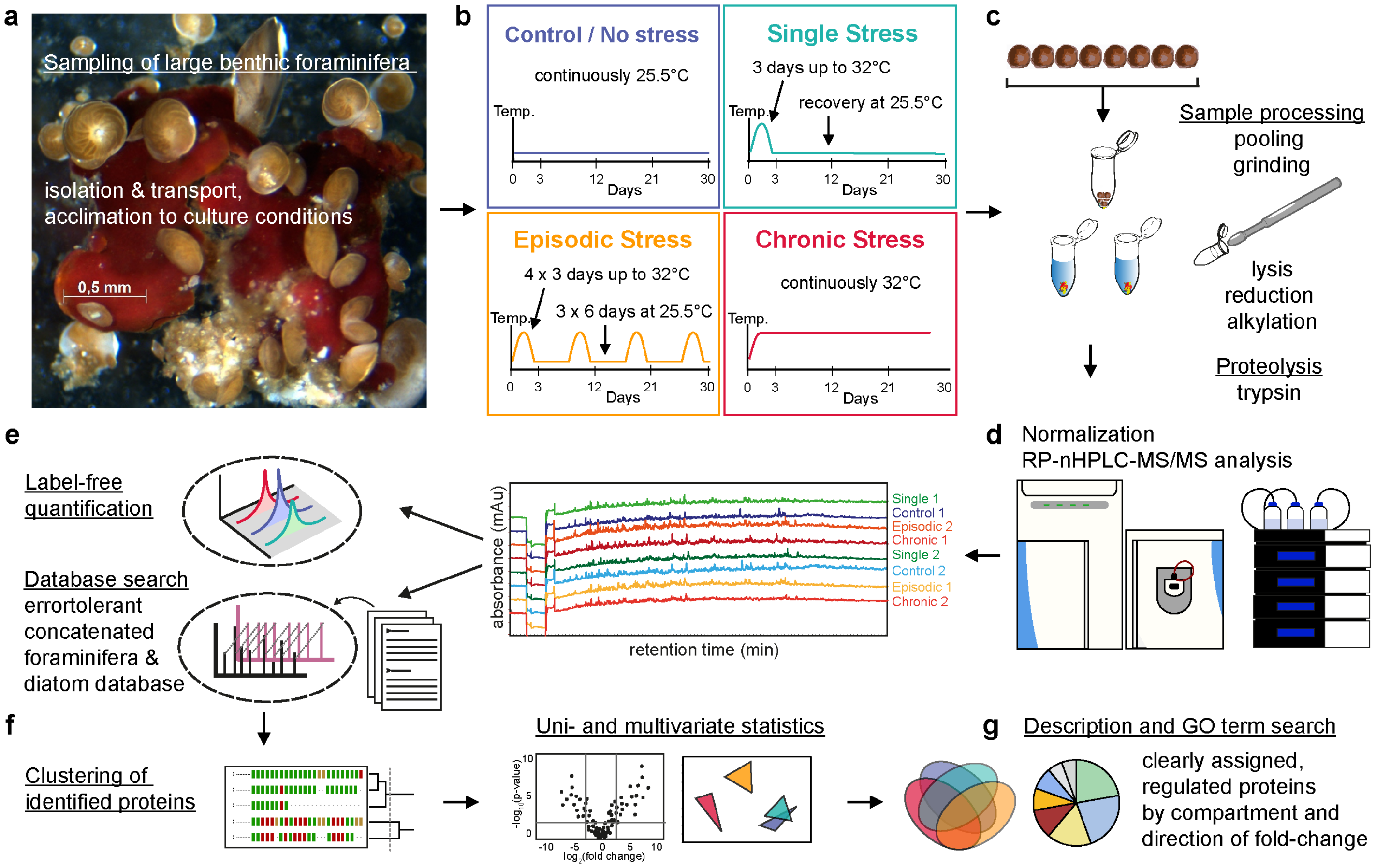

Supplementary Figure S1. Schematic flowchart of the experimental setup and proteome analysis protocol of the thermal stress experiment on *Amphistegina gibbosa*. (a) Specimens were collected from coral rubble in the Florida Keys at 18 m depth, isolated and acclimated for three weeks. (b) Over one month they were exposed to four temperature treatments, with three randomized replicate aquaria per treatment, mimicking different thermal stress scenarios. (c) From each treatment, 8 specimens were pooled, their protein content was extracted and clarified. Before protein digestion by trypsin, their concentrations were measured and samples were cleaned. (d) First, sample amounts of the desalted peptides were normalized by total ion current and then analysed on a LC-MS/MS system. (e) Label-free quantification and protein identification as well as assignment to either host or symbiont compartment was followed by (f) homology-based protein grouping, calculation of protein cluster abundances and determination of statistically significant changes. (g) All protein clusters that were clearly 'regulated' were further characterized by annotation of protein descriptions and gene ontology terms.

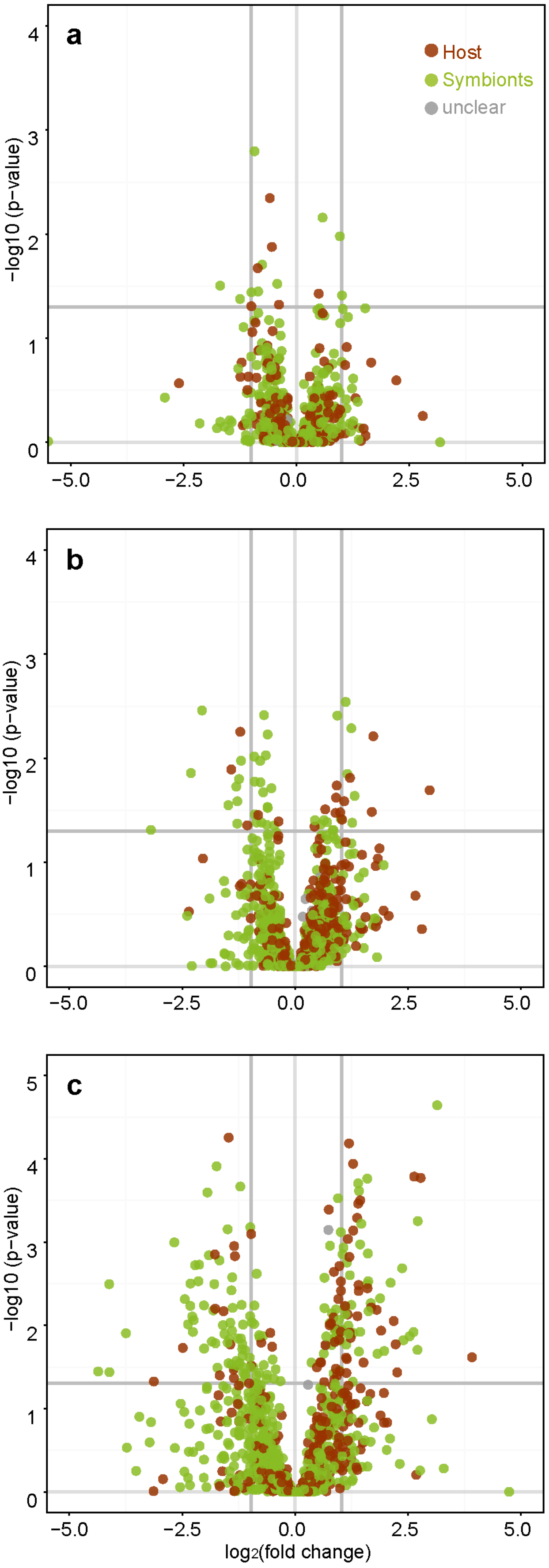

Supplementary Figure S2. Volcano plots of $\log_2$ fold changes versus –log10(*p*-values), representing the probability that the protein is significantly regulated, of all identified proteins in *Amphistegina gibbosa* response to (a) a single stress event, (b) episodic stress events, (c) and chronic stress (please note the different scale of the y-axis), compared to the control. Proteins above –log10(0.05) = 1.301 are considered significantly regulated where fold changes of above $-\log_2(2) = 1$ specify increased and below $-\log_2(0.5) = -1$ specify decreased abundances.

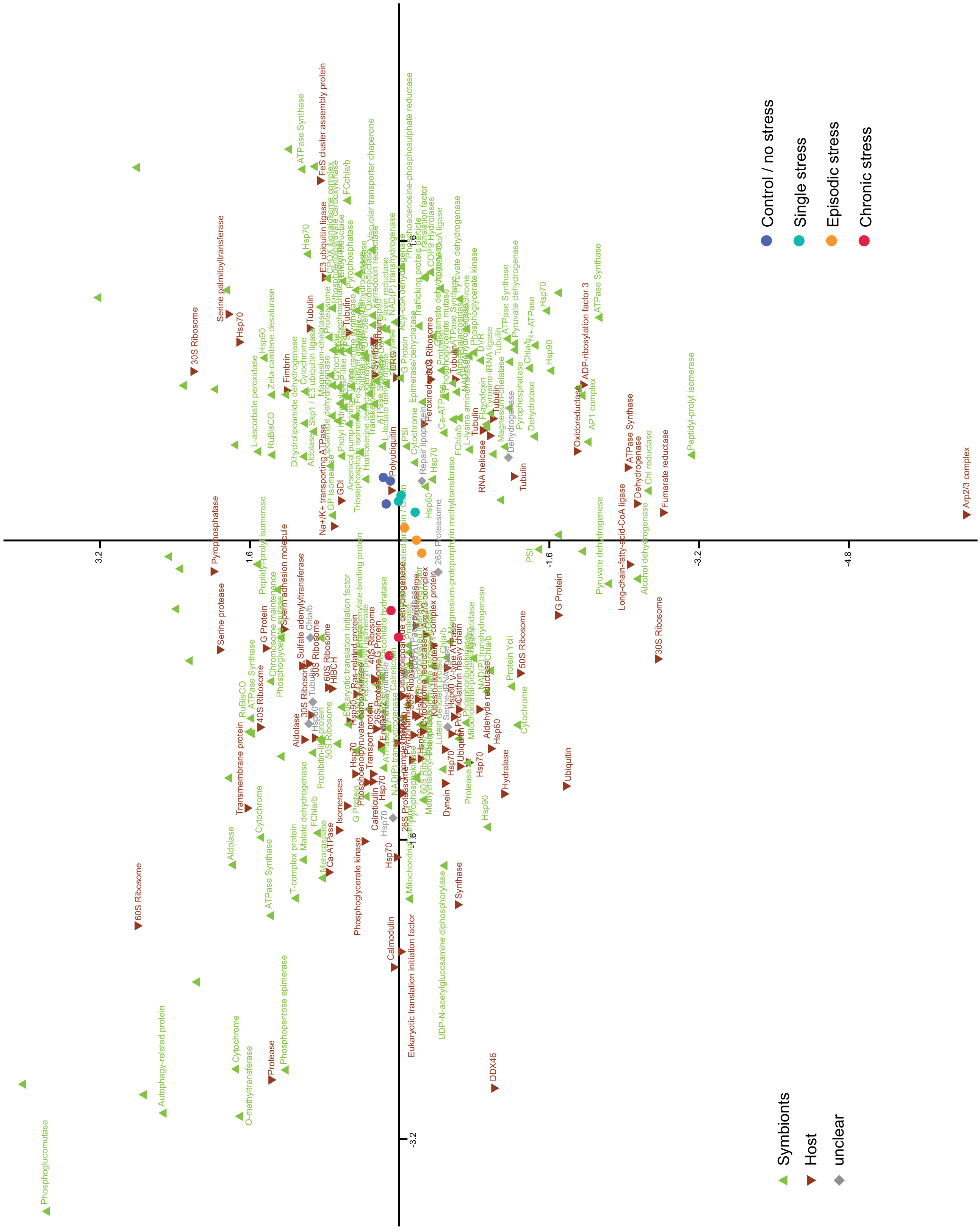

Supplementary Figure S3. Correspondence analysis of relative protein abundances of all 294 regulated proteins in *Amphistegina gibbosa* in response to a single short-term stress event (turquoise), episodic stress events (orange) or chronic thermal stress (red) compared to the control (blue), showing the distribution and descriptions of proteins (host = brown inverse triangles, symbiont = green triangles, unclear/both = grey diamonds) that characterize the directional changes between treatments with protein abundances as scaling variable. 65.8% are explained by variation along axis 1 and 11.2% by axis 2.

Supplementary Table S1. Symbiont-associated proteins that significantly changed in abundance in any of the treatments compared to the control.[a] Row colors indicate functional categories based on GO terms: purple = protein quality control and folding, dark blue = metabolism, green = photosynthesis, orange = biosynthesis and cell cycle, rose = proteolysis and autophagy, light blue = signalling, grey = transport. # = number of proteins in the cluster, UP = number of unique peptides. Log_2 fold changes are indicated by color (red = increase, blue = decrease). Significant results of Tukeys post hoc test are marked by asterisks: (*) *p*-value ≤ 0.05, (**) *p*-value ≤ 0.01 and (***) *p*-value ≤ 0.001.

Consensus Protein Description[a]	#	UP	Log_2 fold change single	episodic	chronic
ATPase synthase subunit beta	1	1	1.01*	1.16*	0.00
26S proteasome regulatory subunit	1	2	1.14	1.32*	0.75
peptidyl-prolyl cis-trans isomerase	1	3	0.69	1.24**	0.57
arginine biosynthesis protein ArgJ (mitochondrial)	1	2	0.96	1.12**	0.53
Clp protease A/B family / chaperonin	1	2	0.38	1.01*	1.13*
Small GTPase superfamily	2	2	0.77	1.30	3.14***
Probable serca-type calcium ATPase	1	1	-0.73	0.78	2.71***
band 7 domain-containing	1	1	-0.17	-1.24	2.71*
vacuolar transporter chaperone domain-containing	1	1	0.71	-1.87	2.62*
alcohol dehydrogenase	1	1	-1.13	-0.11	2.53*
member of the Clp large regulatory subunit	1	1	-1.07	1.03	2.40*
phosphomannose mutase	1	1	-0.12	-0.34	2.37**
histidinol dehydrogenase	1	1	0.66	1.09	2.11**
Tim10/DDP family zinc finger	1	1	0.39	0.05	1.97*
hypersensitive-induced response 1	1	2	-0.32	-0.02	1.88**
heat shock Hsp90	1	3	0.77	1.26	1.82*
arsenical pump ATPase	1	1	-0.27	0.54	1.68**
short chain acyl-CoA dehydrogenase	2	2	-0.01	0.89	1.62**
PP / NAD(P)-binding domain	1	1	0.27	0.77	1.62**
calreticulin	2	5	0.25	0.23	1.61**
heat shock protein hsc70	1	2	-0.11	0.43	1.60***
heat shock 70	2	16	0.34	0.78	1.59**
small GTPase superfamily	1	2	0.48	0.85	1.47***
aconitase hydratase 2	2	5	0.47	0.80	1.44*
delta-aminolevulinic acid dehydratase / porphobilinogen	2	9	-0.23	-0.12	1.44**
heat shock protein/chaperone Hsp70	5	52	0.29	0.70	1.42***
prohibitin-like protein	1	2	0.67	0.55	1.41*
phosphorylated CTD-interacting factor 1	1	1	-0.17	0.39	1.39***
phosphoenolpyruvate carboxykinase	1	1	0.27	0.42	1.28*
tyrosyl-tRNA synthetase	1	1	0.31	0.57	1.28*
methylmalonyl-CoA mutase	2	3	0.46	0.90	1.25**
glyceraldehyde-3-phosphate dehydrogenase	6	17	0.50	0.70	1.21**
cyclin dependent kinase	1	1	0.54	0.34	1.18*
autophagy-related Atg8, ubiquitin-like	2	3	-0.41	-0.21	1.18*
protein of unknown function DUF760	1	1	0.28	-0.25	1.15**
canine-like Rab-type small GTPase	1	2	0.26	0.37	1.11**
plastid lipid-associated protein	1	1	0.51	0.51	1.11**
isochorismatase hydrolase	1	1	-0.06	0.32	1.07**

chaperone protein dnaK / Hsp70-type	4	34	0.37	0.94	1.05**
dihydrolipoyl dehydrogenase	4	7	0.61	0.83	1.03*
NAD-dependent malic enzyme	1	1	-0.05	0.50	1.01*
luminal binding protein / Hsp70 family	1	3	0.03	0.48	1.01***
cyclophilin-type peptidyl-prolyl isomerase	1	2	0.45	0.85	1.01*
LOV domain-containing protein	1	1	-1.25*	-0.69	-1.03
fucoxanthin chlorophyll *a/c*	1	1	-1.69*	-0.55	-1.30
glycoside hydrolase, family 65	1	1	-0.56	-1.20*	-0.14
glycoside hydrolase, family 65	1	2	-0.85	-1.24*	-0.25
catalytic activity	2	3	-0.65	-1.28*	0.02
histidine phosphatase superfamily, clade-1	1	1	-0.94	-1.28*	-1.23*
biotin- / acetyl-CoA carboxylase	1	2	-0.66	-1.30*	-1.81**
NADH dehydrogenase subunit 11 / RuBisCO small chain	1	1	-0.33	-1.48*	-4.12**
phospholipid methyltransferase / phosphoglycerate kinase	1	1	-0.29	-2.06**	-6.57***
HopJ type III effector protein	1	1	-1.17	-2.31*	-2.02*
aldose 1-/glucose-6-phosphate 1-epimerase	1	1	-1.01	-3.19*	-4.36*
phosphoglycerate mutase / chlorophyll *a/b* binding	1	2	-0.31	-0.50	-1.00*
plastid lipid-associated protein / fibrillin conserved domain	1	1	-0.35	-0.21	-1.10*
1-hydroxy-2-methyl-2-(E)-butenyl-4-diphosphate synthase	1	5	-0.03	-0.68	-1.11*
phosphoglycerate kinase	4	37	-0.33	-0.57	-1.11*
magnesium-protoporphyrin IX methyltransferase	1	4	-0.23	-0.34	-1.12*
RuBisCO large subunit, ferredoxin-like	1	1	-0.68	-0.91	-1.15*
transketolase / phosphatidyl-N-methylethanolamine/N-methyltransferase	6	70	-0.44	-0.56	-1.15**
protoporphyrin IX magnesium chelatase, subunit H	2	4	-0.33	-0.64	-1.17*
ATP synthase subunit beta	3	46	0.43	0.27	-1.17*
cytosolic class II aldolase	1	2	-0.42	-0.52	-1.17*
cytochrome b6/f complex, subunit IV	1	1	-0.41	-0.90	-1.18**
dihydrolipoamide acetyl transferase	1	4	-0.56	-0.33	-1.20*
structural maintenance of chromosomes protein	1	1	-0.16	0.15	-1.20*
UDP-glucose-pyrophosphorylase/phosphoglucomutase	4	7	-0.43	-0.61	-1.21***
RuBisCO large	6	84	0.35	0.06	-1.23*
photosystem I iron-sulfur center	2	9	-0.66	-0.72	-1.27*
2-oxoacid dehydrogenase acyltransferase	1	1	-0.05	-0.74	-1.28*
RuBisCO small subunit	4	32	-0.17	-0.60	-1.30*
transketolase-like	2	15	-0.32	-0.62	-1.34*
50S ribosomal protein / cytochrome b6	5	10	0.14	-0.22	-1.38**
precursor of dehydrogenase pyruvate dehydrogenase E1	4	15	-0.30	-0.57	-1.39**
V-type proton ATPase subunit	1	1	-1.00	-0.84	-1.40*
cytochrome b6-f complex iron-sulfur subunit	2	12	0.31	-0.03	-1.41*
oxidoreductase / myo-inositol 2-dehydrogenase	2	10	-0.26	-0.74	-1.41**
acetyl-CoA carboxylase	3	31	-0.30	-0.25	-1.44**
phosphoglycerate mutase	1	3	-0.09	-0.95	-1.46*
cytochrome C peroxidase	1	7	-0.24	-0.13	-1.48*
NAD(P)	1	3	0.08	-0.61	-1.49***
cytochrome b6-f complex subunit IV	1	5	0.12	-0.20	-1.49*
dihydrolipamide S-acetyltransferase	1	2	-0.06	-0.44	-1.56*
cation transporting ATPase	2	5	-0.32	-0.52	-1.60*
ferredoxin--NADP reductase / phosphoglucose isomerase	9	44	-0.29	-0.57	-1.67**
dihydrolipoamide acetyl transferase	1	1	-0.23	-0.62	-1.72*

apospory-associated / ferredoxin--NADP reductase	1	3	-0.33	-0.65	-1.74***
fucoxanthin chlorophyll a/c protein	2	26	-0.34	-0.70	-1.76*
ATP synthase / precursor of ATPase gamma subunit	3	7	-0.10	-0.28	-1.79**
clavaminate synthase	1	1	-0.16	-1.06	-1.84*
transhydrogenase / fructose-biphosphate aldolase	1	4	-0.20	-0.58	-1.91**
fructose-1,6-bisphosphate aldolase	2	21	-0.11	-0.77	-1.94***
lutein deficient 1-like protein	1	1	-0.77	-0.85	-2.02**
cytosolic class II aldolase	2	4	-0.45	-0.85	-2.13**
small ribosomal S1 / photosystem	1	3	0.01	-0.31	-2.14**
pyridine nucleotide-disulphide oxidoreductase	1	1	0.07	-0.40	-2.21**
ATP synthase subunit delta / plastid thylakoid	1	2	-0.41	-0.69	-2.25**
alanine dehydrogenase/pyridine nucleotide transhydrogenase	1	2	0.13	-0.51	-2.26*
cytochrome b6-f complex iron-sulfur subunit / RuBisCO	1	1	-0.35	-0.34	-2.28**
glucose-6-phosphate isomerase	2	4	-0.44	-0.74	-2.32**
lipoamide dehydrogenase	1	9	-0.08	-0.59	-2.32**
ATP synthase beta subunit	7	84	-0.12	-0.43	-2.37**
ATP synthase b'	1	8	-0.39	-0.89	-2.44**
ATP sulfurylase	2	14	-0.24	-0.89	-2.68**
lipoamide dehydrogenase	1	1	-0.37	-0.81	-3.74*
HopJ type III effector protein	2	3	-0.06	-1.57	-4.11*

Supplementary Table S2. Host-associated protein that significantly changed in abundance in any of the treatments compared to the control. [a] Row colors indicate functional categories based on GO terms: yellow = cytoskeleton and microtubule-based processes, purple = protein quality control and folding, dark blue = metabolism, orange = biosynthesis and cell cycle, rose = proteolysis and autophagy, light blue = signalling, grey = transport. # = number of proteins in the cluster, UP = number of unique peptides. Log_2 fold changes are indicated by color (red = increase, blue = decrease). Significant results of Tukeys post hoc test are marked by asterisks: (*) *p*-value ≤ 0.05, (**) *p*-value ≤ 0.01 and (***) *p*-value ≤ 0.001.

**Consensus Protein Description**[a]	**#**	**UP**	**Log_2 fold change**		
			**single**	**episodic**	**chronic**
actin-related protein 2 (Arp2/3 complex)	1	3	2.21	2.98*	0.69
methylmalonyl-CoA mutase	1	1	1.08	1.73**	1.24
fumarate reductase	1	3	0.10	1.21*	-0.20
long-chain-fatty-acid-ligase	1	2	0.20	1.09*	0.14
amidohydrolase 2	1	1	0.96	1.70*	1.90*
chaperonin GRoEL / chaperonin 60	1	8	0.63	1.04*	1.40**
DEAD box polypeptide 46	1	1	1.43	2.81	3.92*
caseinolytic peptidase (ClpA/B family)	1	1	0.35	0.75	2.78***
eukaryotic translation initiation factor 4E type	1	2	0.92	1.48	2.64***
prohibitin	1	2	0.95	1.47	2.26*
ubiquitin / ribosomal protein Cep52 fusion	2	2	1.65	1.78	2.23*
hypothetical protein, kinesin-like	1	1	0.14	1.14	2.18**
phosphoglycerate kinase	1	1	0.44	0.77	1.80**
Hsp70	1	1	0.27	0.81	1.68**
trafficking particle complex subunit 3	1	1	0.43	0.55	1.60**
dynein, heavy polypeptide 5	1	1	0.36	0.84	1.51*
Hsp90 family	1	1	0.20	0.75	1.41**
phosphoenolpyruvate carboxykinase	2	4	0.08	0.53	1.40***
malic enzyme	1	1	0.49	0.57	1.38**
chaperone DnaK / Hsp70	2	7	0.38	0.92	1.37***
calreticulin/calnexin	1	3	0.26	0.53	1.29***
pyrazinamidase/nicotinamidase	1	1	0.51	0.67	1.28***
Heat shock protein 70C	1	2	0.21	0.43	1.23*
Rac GTPase / cell division control 42	1	2	0.48	0.58	1.21*
heat shock 60	1	2	0.07	0.67	1.20**
heat shock 70	2	18	-0.22	0.50	1.19***
GTP-binding Ypt1 / small GTPase superfamily	1	4	-0.20	0.36	1.17***
actin family	1	3	0.42	0.59	1.16**
26S protease regulatory subunit 6b	1	1	0.22	0.52	1.14*
aldo-keto oxidoreductase / alcohol dehydrogenase	1	1	0.42	0.90	1.14*
Hsp70	1	2	-0.07	0.67	1.13*
enolase 2	1	12	0.20	0.45	1.11**
ubiquitin hydrolase	1	3	0.36	0.41	1.02**
cytoplasmic dynein light chain	1	2	-0.07	0.10	1.01**
fructose-bisphosphate aldolase	1	5	0.12	-0.11	1.01*
hydrogen-translocating pyrophosphatase	1	1	-1.00*	-0.81	-0.28
serine carboxypeptidase S10	1	1	-0.91	-1.06*	-1.43*
actin family	1	1	-0.86	-1.22**	-1.78**

serine palmitoyltransferase 1	1	1	-0.34	-1.41*	-1.77**
serine carboxypeptidase S10	1	2	-0.46	-0.32	-1.02*
tubulin alpha-3	1	8	-0.40	-0.36	-1.33**
beta-tubulin	5	73	-0.20	-0.05	-1.35**
tubulin alpha-4	2	4	-0.23	-0.27	-1.35**
calponin homology domain	1	1	-0.98	-0.71	-1.36*
beta tubulin	1	2	-0.59	-0.37	-1.47***
coronin	1	1	-0.19	-0.61	-1.58**
tubulin	1	1	-0.41	-0.85	-1.67*
ATPase, dynein-related	1	1	-0.58	-0.99	-2.49*
phosphoethanolamine N-methyltransferase-like	1	1	-0.02	-2.04	-3.13*

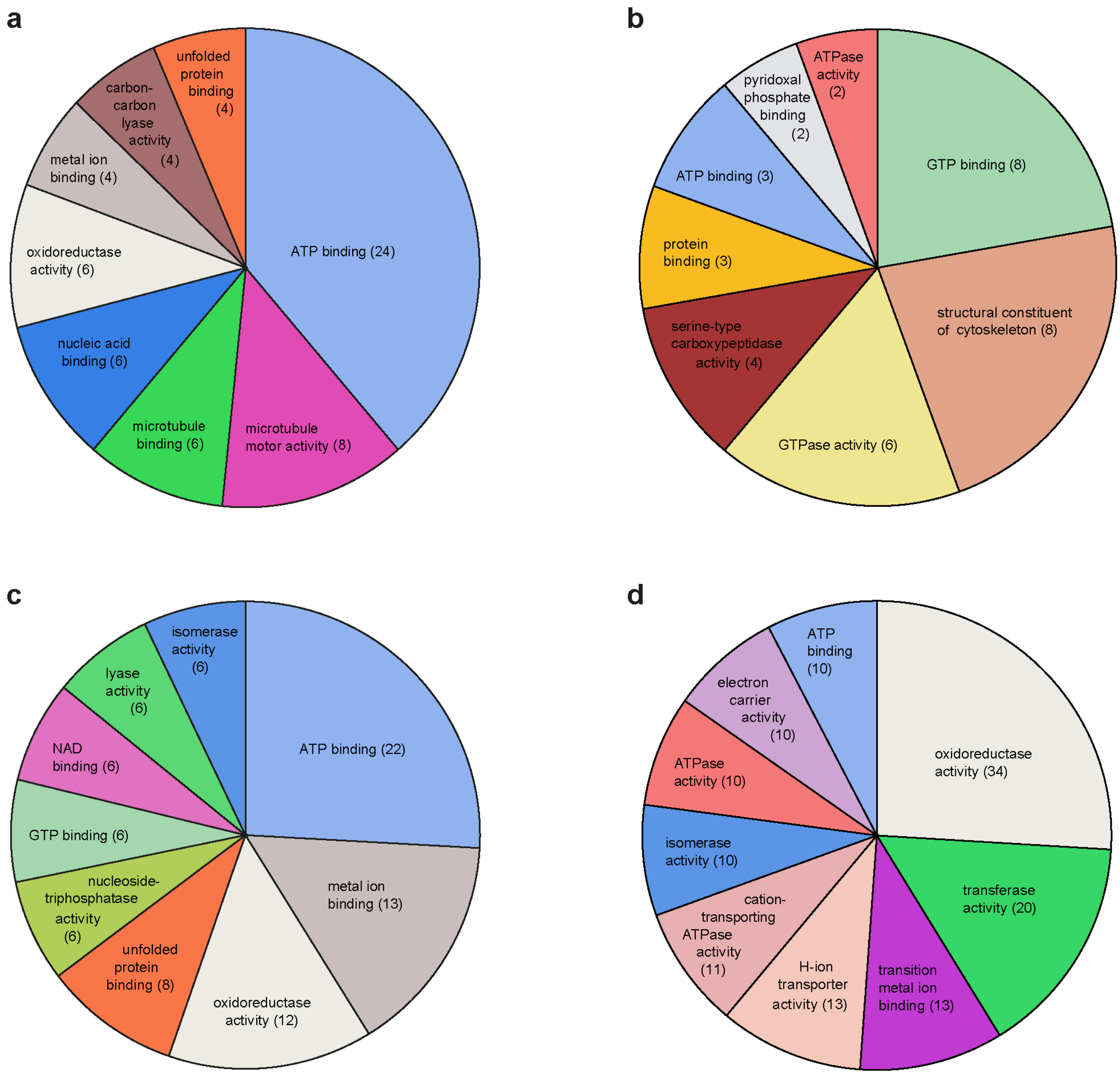

Supplementary Figure S4. Sequence distributions of molecular function annotations of differently abundant proteins in *Amphistegina gibbosa* in response to chronic thermal stress. Protein count charts are arranged by compartment: (a) and (b) are host-associated, (c) and (d) symbiont-associated, as well as by direction of change: (a) and (c) increased, while (b) and (d) decreased compared to the control. Numbers in brackets indicate protein sequence counts of the given gene ontology (GO) function.

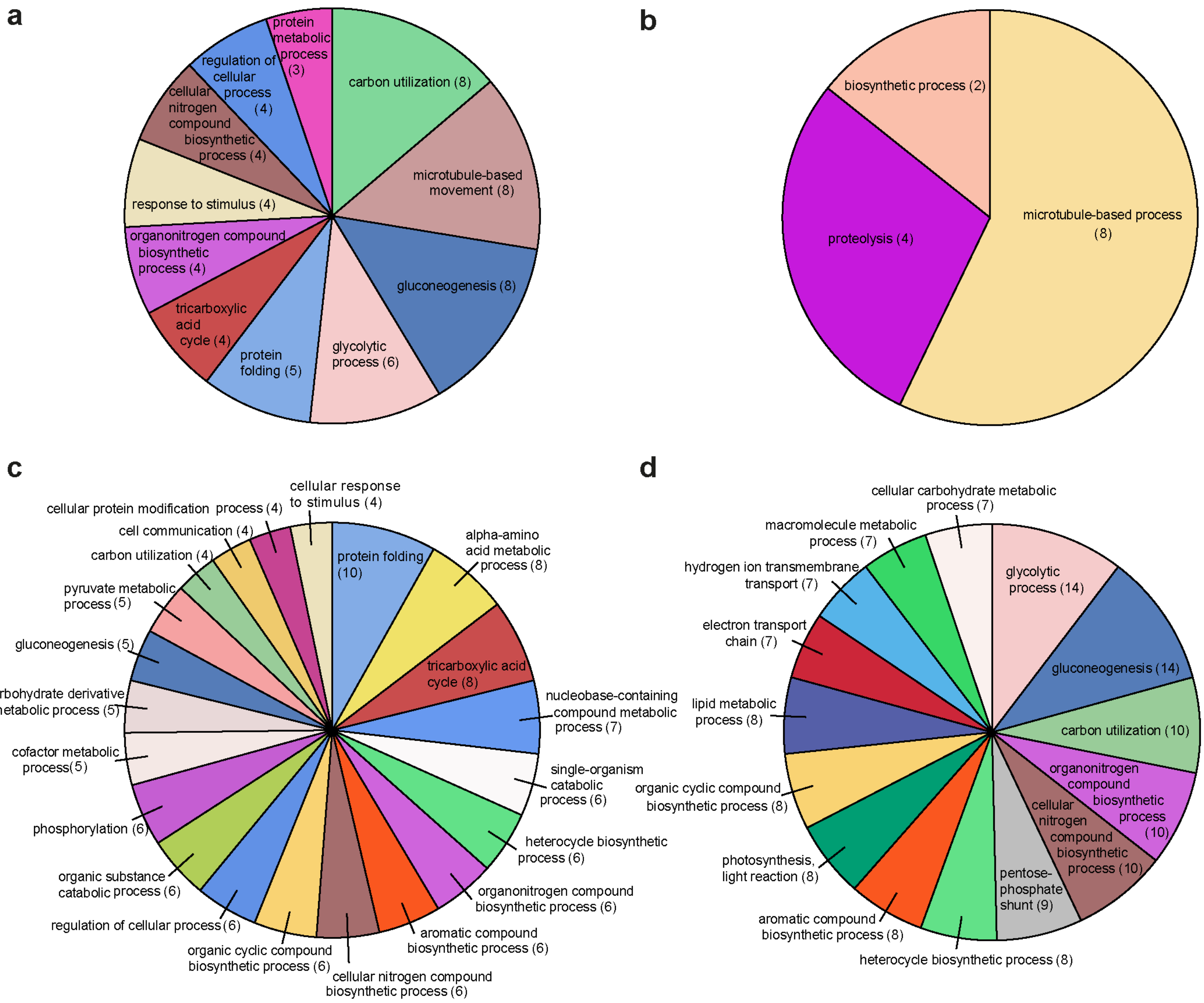

Supplementary Figure S5. Sequence distributions of biological process annotations of differently abundant proteins in *Amphistegina gibbosa* in response to chronic thermal stress. Charts are arranged by compartment: (a) and (b) are host-associated, (c) and (d) symbiont-associated, as well as by direction of change: (a) and (c) increased, while (b) and (d) decreased compared to the control. Numbers in brackets indicate protein sequence counts of the given GO process.

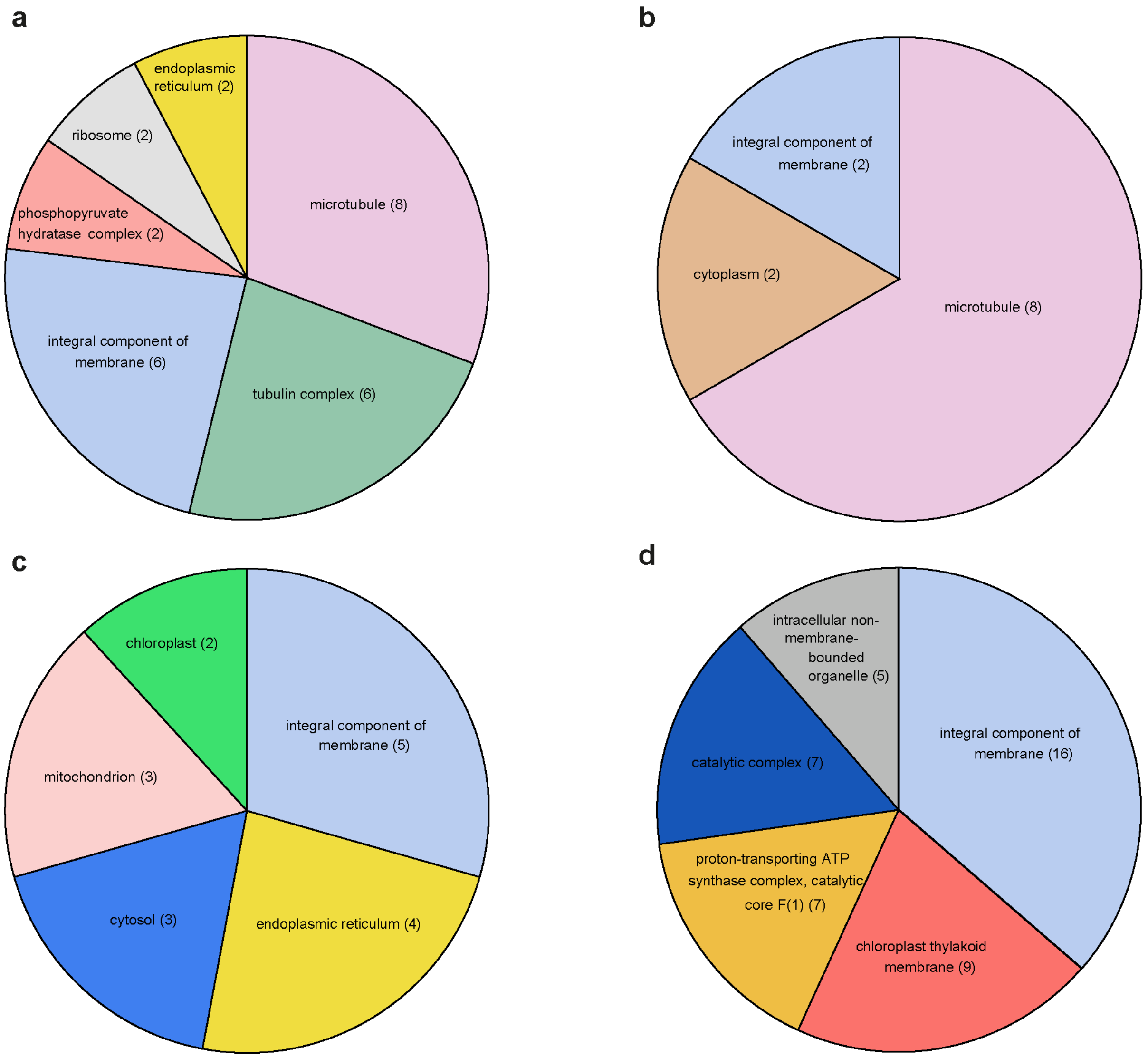

Supplementary Figure S6. Sequence distributions of cellular component annotations of differently abundant proteins in *Amphistegina gibbosa* in response to chronic thermal stress. Protein count charts are arranged by compartment: (a) and (b) are host-associated, (c) and (d) symbiont-associated, as well as by direction of change: (a) and (c) increased, while (b) and (d) decreased compared to the control. Numbers in brackets indicate protein sequence counts of the given GO component.

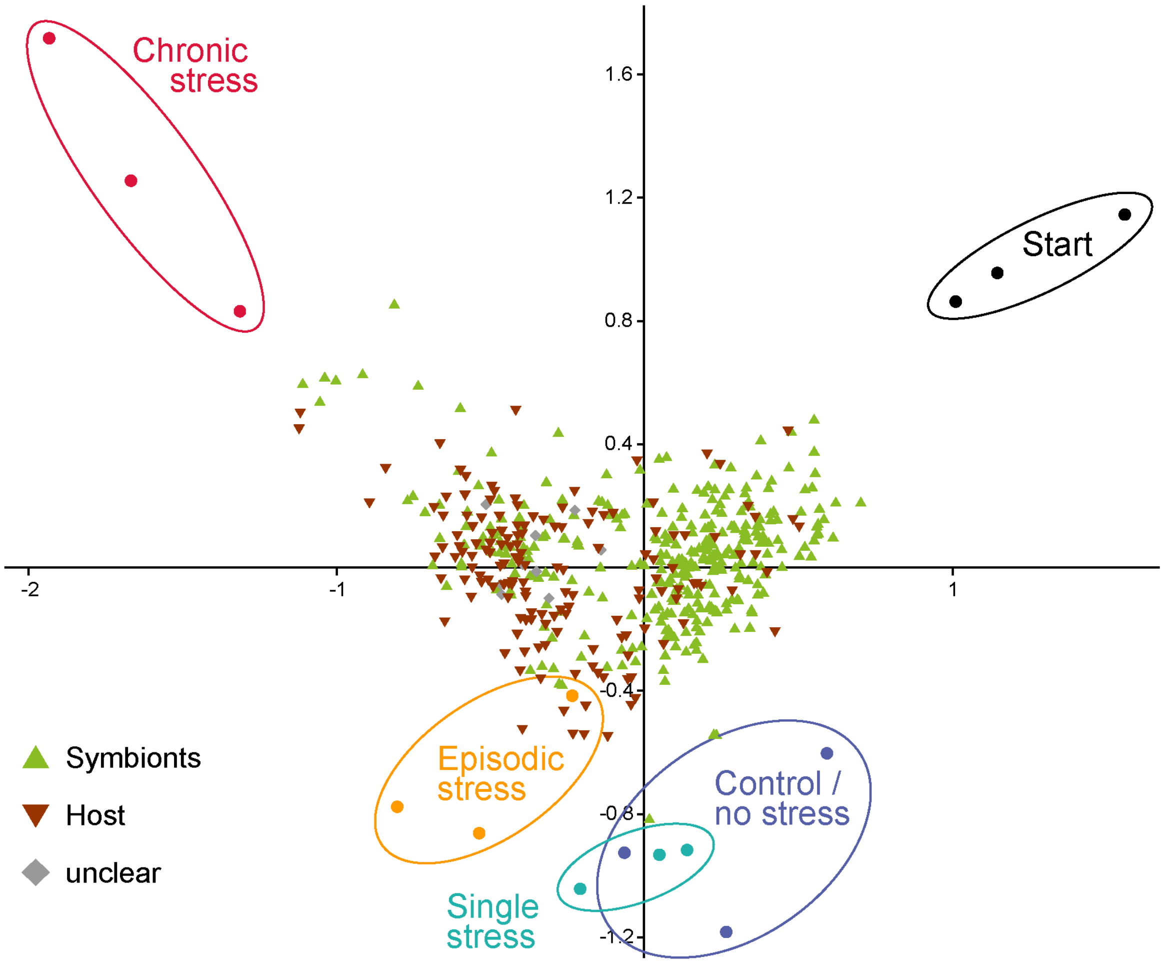

Supplementary Figure S7. Correspondence analysis of relative protein abundances of all 491 regulated proteins in *Amphistegina gibbosa* compared to the start (black) in response to the control treatment (blue), a single short-term stress event (turquoise), episodic stress events (orange) or chronic thermal stress (red), showing the distribution of proteins (host = brown inverse triangles, symbiont = green triangles, unclear/both = grey diamonds) that drive the directional changes between treatments. 55.9% are explained by variation along axis 1 and 17.4% by axis 2.

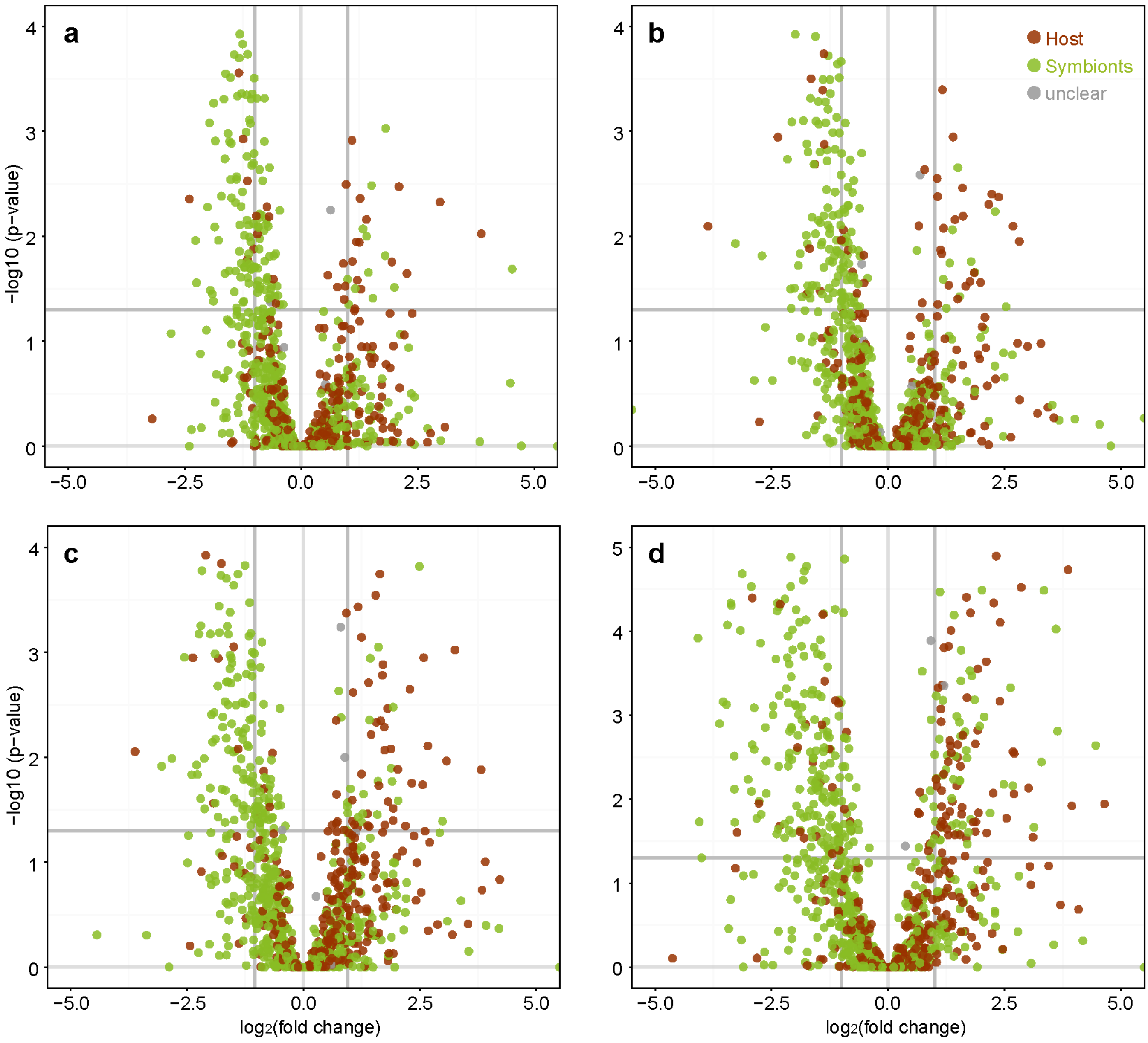

Supplementary Figure S8. Volcano plots of $\log_2$ fold changes versus –log10(*p*-values), representing the probability that the protein is significantly regulated, of all identified proteins in *Amphistegina gibbosa* response to the thermal stress treatments (a) control / no stress, (b) single stress event, (c) episodic stress events, (d) chronic stress (please note different scale of y-axis), compared to the start. Proteins above –log10(0.05) = 1.301 are considered significantly regulated where fold changes of above $-\log_2(2) = 1$ specify increased and below $-\log_2(0.5) = -1$ specify decreased abundances.

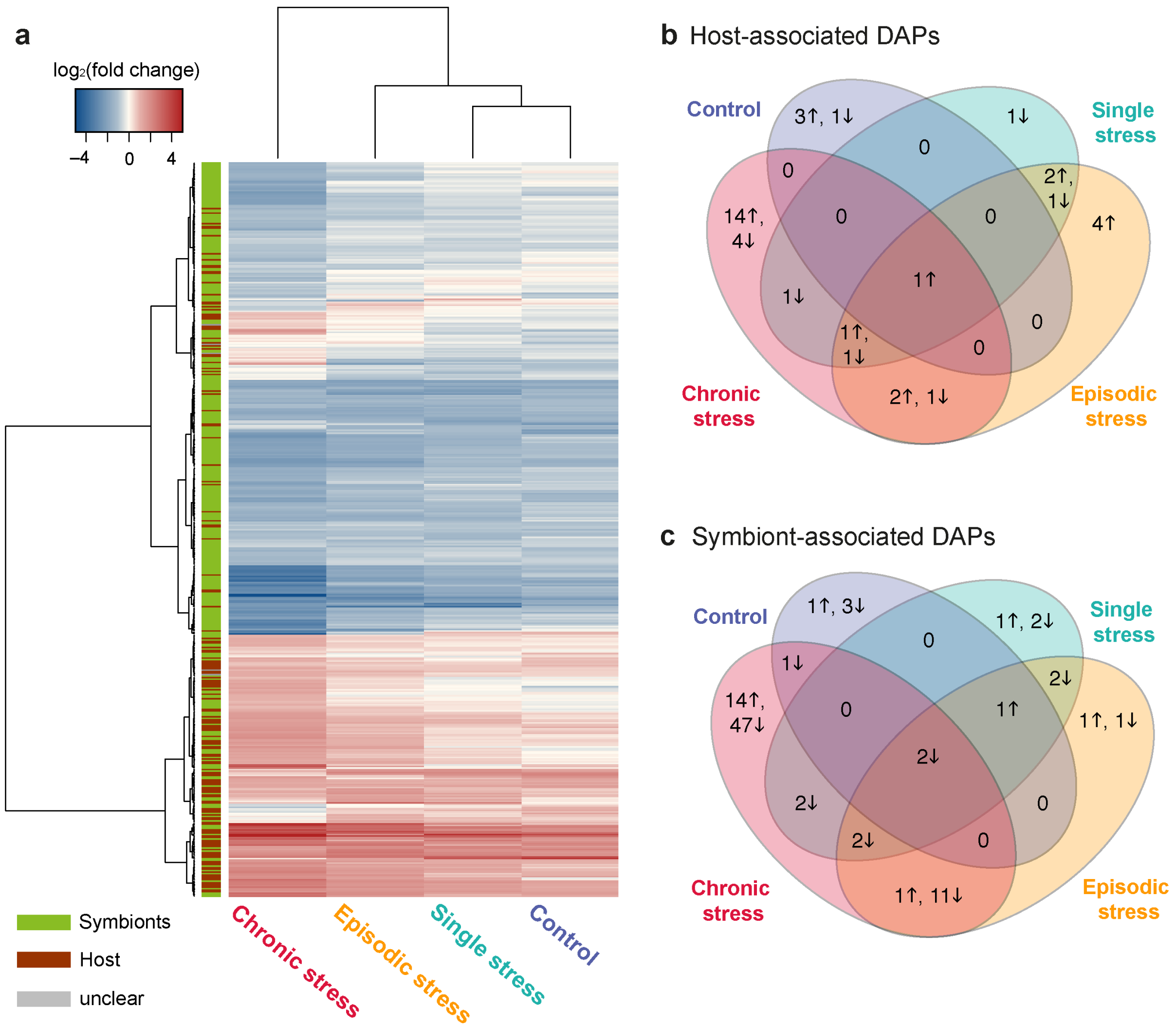

Supplementary Figure S9. The heatmap and hierarchical cluster analysis (Euclidean distance) of all regulated proteins compared to the start (a) illustrates the direction of $\log_2$ fold changes in protein abundances and their distribution among host foraminifera (brown) and symbionts (green). The normalized abundance values, significant *p*-values, and accessions of all regulated protein clusters are reported in Table S3. Venn diagrams of the amount of proteins that significantly changed in abundance in response to control conditions (blue), a single stress event (turquoise), episodic stress events (orange) and chronic stress (red) compared to the control in (b) the host (n = 37) and (c) the symbiont compartment (n = 92). Overlapping areas show protein groups that were equally regulated in more than one treatment. Arrows indicate how many proteins were up (↑) or down (↓) regulated.
